# Mutations of SARS-CoV-2 Structural Proteins in the Alpha, Beta, Gamma, and Delta Variants: Bioinformatics Analysis

**DOI:** 10.2196/43906

**Published:** 2023-07-14

**Authors:** Saima Rehman Khetran, Roma Mustafa

**Affiliations:** 1 Department of Life Sciences Sardar Bahadur Khan Women's University Quetta Pakistan

**Keywords:** virus evolution, influenza and other respiratory viruses, advances in virus research, COVID-19, protein, mutation, genomic, vaccine development, phylogenetic analysis, biochemistry

## Abstract

**Background:**

COVID-19 and Middle East Respiratory Syndrome are two pandemic respiratory diseases caused by coronavirus species. The novel disease COVID-19 caused by SARS-CoV-2 was first reported in Wuhan, Hubei Province, China, in December 2019, and became a pandemic within 2-3 months, affecting social and economic platforms worldwide. Despite the rapid development of vaccines, there have been obstacles to their distribution, including a lack of fundamental resources, poor immunization, and manual vaccine replication. Several variants of the original Wuhan strain have emerged in the last 3 years, which can pose a further challenge for control and vaccine development.

**Objective:**

The aim of this study was to comprehensively analyze mutations in SARS-CoV-2 variants of concern (VoCs) using a bioinformatics approach toward identifying novel mutations that may be helpful in developing new vaccines by targeting these sites.

**Methods:**

Reference sequences of the SARS-CoV-2 spike (YP_009724390) and nucleocapsid (YP_009724397) proteins were compared to retrieved sequences of isolates of four VoCs from 14 countries for mutational and evolutionary analyses. Multiple sequence alignment was performed and phylogenetic trees were constructed by the neighbor-joining method with 1000 bootstrap replicates using MEGA (version 6). Mutations in amino acid sequences were analyzed using the MultAlin online tool (version 5.4.1).

**Results:**

Among the four VoCs, a total of 143 nonsynonymous mutations and 8 deletions were identified in the spike and nucleocapsid proteins. Multiple sequence alignment and amino acid substitution analysis revealed new mutations, including G72W, M2101I, L139F, 209-211 deletion, G212S, P199L, P67S, I292T, and substitutions with unknown amino acid replacement, reported in Egypt (MW533289), the United Kingdom (MT906649), and other regions. The variants B.1.1.7 (Alpha variant) and B.1.617.2 (Delta variant), characterized by higher transmissibility and lethality, harbored the amino acid substitutions D614G, R203K, and G204R with higher prevalence rates in most sequences. Phylogenetic analysis among the novel SARS-CoV-2 variant proteins and some previously reported β-coronavirus proteins indicated that either the evolutionary clade was weakly supported or not supported at all by the β-coronavirus species.

**Conclusions:**

This study could contribute toward gaining a better understanding of the basic nature of SARS-CoV-2 and its four major variants. The numerous novel mutations detected could also provide a better understanding of VoCs and help in identifying suitable mutations for vaccine targets. Moreover, these data offer evidence for new types of mutations in VoCs, which will provide insight into the epidemiology of SARS-CoV-2.

## Introduction

The emergence of SARS-CoV-2 during the early months of 2020 made headlines worldwide. Since then, several new variants of SARS-CoV-2 have emerged and are classified based on their ability to cause a threat to public health in two groups: variants of concern (VoCs) and variants of interest (VoIs) [1]. VoIs are defined as variants with specific genetic markers causing mutations that facilitate virus transmissibility, reduce the accuracy of diagnostic results, and reduce antibody neutralization acquired through natural infection or vaccination [2]. VoCs are associated with the level of virus transmissibility, infection, reduced effectiveness of vaccines and treatment, failure in virus detection, and reduced levels of neutralizing antibodies generated during previous vaccination or infection. The main SARS-CoV-2 VoCs that emerged include the Alpha (B.1.1.7), Beta (B.1.351), Gamma (P.1), Delta (B.1.617.2), and the most recent Omicron variants [2]

The first reported VoC, B.1.1.7 (Alpha variant), was isolated in the United Kingdom in December of 2020 [3,4], which contained a total of 23 mutations [5]. These mutations directly affect the open reading frame (ORF)1ab and ORF8 regions of the spike (S) protein as well as the nucleocapsid (N) protein [6]. The Alpha variant was characterized by substantially higher levels of infectivity and transmissibility. A total of seven mutations were reported in the S protein of this variant, including N501Y, A570D, D614G, P681H, T716I, S982A, and D1118H, along with two deletions (Δ69-70 and Δ145) [7]. In addition, several mutations were identified in the N protein sequence of the Alpha variant, including D3L, P13L, D103Y, S197L, S188L, S93I, I292T, R203K, G204R, S190I, S194L, S202N, S235F, D348H, and D401Y [8].

The second reported VoC of SARS-CoV-2, B.1.351 (Beta variant), was identified during the second wave in Africa in October 2020 [9]. This variant included a total of nine point mutations (L18F, D80A, D215G, R246I, K417N, E484K, N501Y, D614G, and A701V) and three deletions (Δ242-Δ244) in the S protein, but only one mutation in the N protein (T205I) [10,11]. Subsequently, another VoC, P.1 (Gamma variant), was first identified in Brazil at the end of January 2021 [12] harboring 10 mutations in the S protein (L18F, T20N, P26S, D138Y, R190S, K417T, E484K, N501Y, H655Y, T1027I) [2,12] and 3 mutations in the N protein (R203K, G204R/X, P80R) [13,14].

Among the other reported VoCs, B.1.617.2 (Delta variant), the most dominant variant of SARS-CoV-2, was first detected in India during the second wave of the COVID-19 outbreak in April 2021 [15]. The Delta variant harbored 10 point mutations (T19R, G142D, R158G, L452R, T478K, D614G, P681R, and D950N) [2] and two deletions (Δ156, Δ157) in S protein, but the most lethal among them were L452R and P681R [15,16], as isolates carrying these mutations were responsible for most of the deaths in India and other countries. Moreover, Delta acquired several mutations in the N protein, including G18C, D63G, L139F, R203M, G215C, A252S, S327L, D377Y, and R385K [17,18].

In addition to the S and N proteins, all of these reported VoCs comprise the other main structural proteins of SARS-CoV-2, membrane protein (M) and envelope protein (E). Each protein of the virus plays a vital role and also takes part in the replication cycle. The S protein is required for the attachment and amalgamation of the virus to host cell surface receptors, which enables the virus to enter the host cell [19]. The main function of the N protein is to form the nucleocapsid by binding to the RNA genome of the virus, which has unique properties compared to the other proteins. The shape of the virus envelope is developed with the help of M protein, which is present in abundance inside the virus and also facilitates interactions with other viral proteins as well as in organizing the assembly of proteins. E protein is the smallest SARS-CoV-2 protein, whose function remains somewhat mysterious. During replication, E protein is abundantly expressed in the host cell, whereas only a small portion of the protein is incorporated into the virus envelope [20]. Almost all of the major structural proteins possess mutations at the receptor-binding domain (RBD) and N-terminal domain sites [21], and they have one mutation in common (N501Y) except for the Delta variant [21].

Recent studies have shown that several mutations are responsible for the spread and lethality of SARS-CoV-2, with more than 10 SARS-CoV-2 variants reported to date that are categorized as either VoCs or VoIs [3]. However, it remains unclear how these sequences are mutating during transfer of the virus from person to person. To answer this question, a total of 127 full-length amino acid sequences of SARS-CoV-2 isolates from 14 countries submitted to NCBI up to July 15, 2021, were retrieved to investigate and identify amino acid substitutions in SARS-CoV-2 lineages and their mutational pattern in major structural proteins.

In this study, we used bioinformatics methods to identify nonsynonymous mutations in the S and N proteins of the four main VoCs of SARS-CoV-2 and to determine how they affect the structure and functional dynamics of the virus. This analysis will help to better understand the epidemiology of SARS-CoV-2 and its emerging VoCs, which might ultimately identify suitable mutations as new vaccine targets.

## Methods

### Data Source

On the basis of a high predominance rate, the data were collected from isolates reported in 14 countries (Pakistan, Turkey, China, Iran, Morocco, United States, United Kingdom, France, Italy, Spain, India, Japan, Egypt, and Russia). A total of 127 nucleotide sequences of SARS-CoV-2 were retrieved from the National Center for Biotechnology Information (NCBI) Virus SARS-CoV-2 Data Hub [22] along with their major structural proteins (Table 1).

SARS-CoV-2 sequences for the four respective proteins (S, N, M, and E) were downloaded in FASTA format. Reference sequences were also considered for data comparison. A total of 127 amino acid sequences were obtained for analysis, which were converted into nucleotides using the online reverse translation tool Sequence Manipulation Suite.

**Table 1:** Number of isolates corresponding to the sequences retrieved from various nations.

Variant of SARS-CoV-2	Countries	Number of isolates
Reference sequence	China	1
β-Coronavirus isolates	United States	5
Alpha variant	Pakistan, Turkey, China, Iran, Morocco, United States, United Kingdom, France, Italy, Spain, India, Japan, Egypt, Russia	78
Beta variant	United States, Italy, Spain, France, India	20
Gamma variant	United States, Italy, Pakistan, Spain, Egypt, India	15
Delta variant	India, Egypt, United States, Spain	10

### Quality Profiling for Sequence Selection and Phylogenetic Analysis

Quality profiling for sequence selection was performed to differentiate between countries according to the epidemic record. This study included four types of SARS-CoV-2 variants taking into account their S and N proteins. Data were compared by constructing phylogenetic trees for each protein. All other types of SARS-CoV-2 variants and their respective proteins were excluded from the analysis.

The phylogenetic tree was constructed from 127 sequences of the major proteins along with the corresponding reference protein sequences (S protein: YP_009724392; N protein: YP_009724393; M protein: YP_009724397; and E protein: YP_009724390). Protein sequences of β-coronavirus strains were selected as outgroups: human coronavirus (hCoV)-NL63 (YP_003767), hCoV-229E (NP_073551), hCoV-OC43 (YP_009555241), Middle East Respiratory Syndrome (MERS; YP_009047204), and SARS (NC_004718).

Multiple sequence alignment was performed and phylogenetic trees were constructed by the neighbor-joining method with 1000 bootstrap replicates using MEGA (version 6) [23]. The FASTA file was computed with a gap-opening penalty of 15 and gap-extension penalty of 6.66, maintaining a delay divergent cutoff of 30%. Amino acid substitutions that were unique to SARS-CoV-2 were identified by visual inspection of the alignments.

### Mutation Identification With MultAlin

For the detection of widespread nonsynonymous mutations in the S, N, M, and E proteins, amino acid sequences were analyzed using MultAlin (version 5.4.1) [20] and each mutation was recorded separately.

This tool enabled identifying the exact location of the mutation in the genome sequence of each strain by providing the position of the mutated site.

### Ethics Considerations

This study was based on analysis of secondary data that are publicly available at NCBI [22] and did not require any ethical approval.

## Results and Discussion

### Mutation Hotspots in the S Protein of SARS-CoV-2 Variants

The main VoCs of SARS-CoV-2 all contain the four major structural proteins S, N, M, and E, and numerous studies have elucidated similarities and differences among the viral genomes and their proteins using different types of bioinformatics tools [23]. Among the isolates of the 14 countries considered in this study, strong evidence was found for occurrence of the D614G mutation (see Multimedia Appendix 1), indicating replacement of the amino acid aspartic acid (D) with glycine (G) at position 614 in the sequence. The D614G mutation affects the interaction with the host receptor angiotensin-converting enzyme 2 (ACE2), resulting in greater stability and the ability to transmit more efficiently, although binding of the mutant was not as competent as compared to the normal binding of the viral protein [24]. The majority of the Pakistan isolates (MW421982–92) also carried the D614G mutation along with some unreported additional mutations, including P26L, D80Y, S813N, Q1207H, D1163Y, and T1117I (see Multimedia Appendix 1). Two of the Egyptian isolates (MW533286, MW533289) displayed unique mutations (Q23X, S12X, Q677X, and P681X), where the amino acids glutamine (Q), serine (S), and proline (P) were replaced with an unknown amino acid (X) at different positions. These mutations might be important for the future study of the mechanism underlying virus lethality. A much higher rate of mutations along with D614G was observed in the UK isolate MT906649, with a series of novel mutations (T22X, P25X, G142X, Y144-5X, S735X, K1191X) identified in which the existing amino acids tyrosine (Y), threonine (T), proline (P), lysine (K), and glycine (G) were substituted to result in a change of the conformation of S protein (see Multimedia Appendices 1 and 2).

Another mutation of concern identified in S protein was A570D, in which alanine (A) was replaced by aspartic acid (D) at position 570, which was found to co-occur with a Δ145 deletion and three other mutations: T716I, where tryptophan (T) was replaced by isoleucine (I) at position 716; S982A, where serine (S) was replaced by alanine (A) at position 982; and D1118H, where aspartic acid (D) was replaced by histidine (H) at position 1118 (Multimedia Appendix 1). The mutations A570D, T716I, S982A, and D1118H were a result of a series of accumulated mutations, which collectively increased the lethality and transmissibility of the virus [25]. These mutations were observed in isolates from the United States, India, Italy, and Spain simultaneously; nevertheless, the US isolates (MW725912, MW725900, MW725904, MW725907, MW712865, MW712862, MW712864, MW725917, and MW725924) also harbored the mutation G72W, in which glycine (G) was replaced by tryptophan (W) at position 72W, along with D614G although the effect of this mutation remains unknown. The co-occurrence of the mutation A570D with D614G and S982A was also observed in some isolates of Italy (MW491232, MW711159), the United States (MZ311101 and MW725906), and India (MW600456), with no other novel mutations identified in these cases (Multimedia Appendices 1 and 2). The mutations A570D, D614G, and S982A correspondingly help in minimizing contact between the individual trimeric spike promoter chains, thereby promoting increased cleavage between the S1 and S2 domains of S protein to consequently enhance the host fusion capability while rearranging the overall dynamic structure of the virus [26].

The third most prominent mutation identified was N501Y, in which asparagine (N) was replaced by tyrosine (Y) at position 501 of the S protein (Multimedia Appendix 1). The transmissibility of the virus harboring the N501Y mutation (located at the receptor-binding motif) increased by 70%-80% and this mutation also improved the binding affinity of the virus onto host cells [27]. This mutation in combination with 7 other mutations (A570D, P681H, T716I, S982A, D1118H, and Δ69-70, Δ145) were termed to be “mutations of major concern” [27,28] and were consistently detected in isolates from the United States, India, Italy, and Spain. The deletion of histidine at position 69 (Δ69) and valine at position 70 (Δ70) also evolved in other variants (Multimedia Appendix 1) and are considered to be responsible for increasing the transmissibility as well as infectivity of the virus, along with causing S gene target failure, resulting in nondetection of the virus [7,29]. Another deletion of tyrosine at position 144 (Δ144) was considered to be responsible for changing the conformation of the S protein’s surface, thereby facilitating evasion of host immunity and increasing infection [30]. Apart from these mutations, deletions at position 85-89 (Δ85-Δ89) in a Spanish isolate (MW715071) along with other unique mutations of S protein, such as V90T (in which valine is replaced by threonine at position 90), A93Y (in which alanine is replaced by tyrosine at position 93), and D138H (in which aspartic acid is replaced by histidine at position 138), were also observed (Multimedia Appendices 1 and 2). Although the specific function of these mutations remains unknown, their identification and further analysis may help to better understand virus structure and lethality.

Additionally, the trio mutations A220V (alanine replaced by valine at position 220), ORF10 V30L, and Spike A222V, were identified in the S and N proteins of Spanish isolates (MW715068-MW715080). These mutations formed different types of clades when combined with other mutations [31], although the A220V mutation was identified with no additional mutations from the reported data. Furthermore, some of the main mutations included in South African variants were L18F (leucine replaced by phenylalanine at position 18), D80A (aspartic acid replaced by alanine at position 80), D215G (aspartic acid replaced by glycine at position 215), R246I (arginine replaced by isoleucine at position 246), K417N (lysine replaced by asparagine at position 417), and E484K (glutamic acid replaced by lysine at position 484), along with N501Y, D614G, and A222V. The mutations K417N, E484K, and N501Y located in the RBD help the virus in binding to the ACE2 receptors of host cells [9,10] A recent study also reported that the E484K mutation might alter the conformation of S protein, thereby affecting the neutralizing capability of the antibody response in host cells, as cases of reinfection were also increased in patients with isolates harboring the E48K mutation at the peak (ie, the majority of the isolates possessed the E484K mutation) during mid-2021 [27]. Several studies have also reported the E484K mutation as a major cause of decreased effectiveness of current vaccines [27,32]. The mutations N501Y and E484K along with L18F and K417T/N are considered to decrease ACE2 binding affinity [33] and were reported in isolates from Italy (MW642250 and MW642248) and the United States (MZ320527). Some of the mutations of the Alpha and Gamma variants, such as N501Y, D614G, E484K, A701V, and N501Y, were also observed in isolates from Italy and the United States (Multimedia Appendices 1 and 2).

As the variants continued to spread across different regions, another VoC emerged in Spain toward the end of 2020. This variant possessed an exceptional mutation, A222V (alanine replaced by valine at position 222), in the S protein (Multimedia Appendix 1). The mutation A222V alone had no direct impact on transmissibility of the virus, in contrast to the effect of D614G [34]; however, in combination with other Beta variant mutations such as L18F, D80A, K417N, E484K, N501Y, A701V, D215G, and deletions at position 242-244 (Δ242-Δ244), A222V causes a severe hindrance in antibody binding [29]. We found these mutations combined with D614G in Spanish isolates (MW715072 and MW715075). A new type of deletion (Δ139-Δ144) was also observed in two isolates from Spain (MW715068 and MW715078) along with the L18F, A222V, and D614G mutations (Multimedia Appendices 1 and 2).

In addition to the Alpha and Beta variants, another VoC was the Brazil variant, which consists of mutations almost identical to S protein mutations of the Beta variant (N501Y, E484K, A701Y) except for the K417T mutation, where lysine (K) was replaced by threonine (T) at position 417, also causing a decrease in ACE2 binding affinity [33]. The dominance of these mutations in many VoCs that play an important role in ACE2 binding affinity during viral attachment [33] might also increase the chances of reinfection [35]. These mutations occurred in isolates from Italy (MW642250, MW642248, MW711159, and MW491232), the United States (MZ320527), and France (MW580244) (Multimedia Appendices 1 and 3).

As compared to other VoCs, the Delta variant was of major concern, which consists of four types of signature mutations: L452R, T478K, D614G, and P681R. The P681R mutation, in which proline (P) was replaced by arginine (R) at position 681, increased the rate of the cleavage process in S1 and S2 subunits (at the furin cleavage site), facilitating virus transmissibility [33,36]. A famous virologist at Cornell University in New York stated that “This little insert (P681R) sticks out and hits you in the face” [36]. The P681R mutation was considered to be responsible for the rapid spread of SARS-CoV-2 around the globe [36]. These signature mutations (L452R, T478K, D614G) were observed in isolates from Egypt (MW533290), India (MZ310590 and MZ310591), and Spain (MW715070) (Multimedia Appendix 4). L452R is the only S protein mutation that clasps the virus with the host cell surface, facilitating injection of the viral genetic material into host cells [4]. The L452R mutation was identified in isolates from the United States (MW725963) and Spain (MW715074) along with D614G, covering more than 90% of variants that emerged since 2020, conferring the virus with increased replication and infectivity abilities [24,36] (Multimedia Appendices 1 and 4).

These data demonstrated that the UK variant 20I/N501Y.V1 derived from lineage B.1.1.7 and the Brazil variant 20J/501Y.V2 derived from lineage B.1.351 (termed P.1) consisted of several mutations at specific points of the nucleic acid sequence, causing several physical changes as well as functional changes affecting virus lethality.

### Mutation Hotspots in N protein of SARS-CoV-2 Variants

Among the other structural proteins of SARS-CoV-2, N protein, which is known to be more stable and conserved than other proteins, consists of three domains: the N-terminal domain, serine/arginine-rich linker region, and C-terminal domain [37]. The function of N protein is to make the nucleocapsid for the virus by binding to its RNA genome [38]. A few mutations have been observed in the N protein of the Alpha variant, including D3L (aspartic acid replaced by leucine at position 3), R203K (arginine replaced by lysine at position 203), G204R (glycine replaced by arginine at position 204), S194L (serine replaced by leucine at position 194), and S235F (serine replaced by phenylalanine at position 235), along with a single mutation of the Beta variant (T205I, in which threonine is replaced by isoleucine at position 205). Few mutations were observed in the Gamma variant, including R203K and G204R/X. Moreover, the N protein of the Delta variant exhibited the mutations R203M (arginine replaced by methionine at position 203), G204R, and D377Y (aspartic acid replaced by tyrosine at position 377).

The tetrad mutations D3L, R203K, G204R, and S235F were observed in isolates from the United States (MW712861-64, MZ311101, and MW725900-24), Italy (MW711159, MW491232), India (MW600456-58), and Spain (MW715071). The extraordinary sequence of Spain (MW715071) also possesses a unique deletion at position 209-211 whose function has not yet been reported. This sequence (MW715071) is termed extraordinary because of its unique genotypic characteristics along with the presence of a high number of unique mutations that had not been previously identified, including V90T, A93Y, D138H, and G212S (glycine replaced by serine at position 212), and deletions at positions 85-89 of S protein as well as at positions 209-211 of N protein (Multimedia Appendices 2 and 5).

Mutually, R203K-G204R mutations were observed in the serine/arginine-rich linker region (responsible for cellular processes such as the cell cycle and characterized by high flexibility) of N protein, which also affect virus assembly [11,39]. Additionally, R203K-G204R mutations belong to the Alpha (along with D3L and S235F) and Gamma variants of SARS-CoV-2 (also expressed as G204R/X) [39,40]. The function of the G204X mutation can be considered ambiguous at present, because the specific amino acid replacing glycine at position 204 is unknown. Additionally, the amino acid substitutions R203K-G204R were identified in various isolates from 11 regions. The Egypt isolates (MW533286, MW533289) also possess the peculiar mutations G212X and G25X, in which glycine is replaced by an unknown residue at positions 25 and 212 (Multimedia Appendix 2), whereas a Pakistan isolate (MW422070) harbors the mutations R203K, G204R, and D614G of the Alpha variant along with an additional mutation A152X, where alanine (A) is replaced by an unknown amino acid residue (X) at position 152 (Multimedia Appendix 3). The mutations R203K, G204R, and D614G also increase viral infectivity due to a higher replication rate; thus, the presence of the dual mutation R203K/G204R in N protein along with the D614G and N501Y mutations of S protein result in an overall increase in the severity of disease and viral infectivity in the host body [14].

The R203K/G204R and N501Y mutations were also associated with disease severity, infectivity of the virus, and an increase in the mortality rate of host cells [41,42]. The combinations of R203K/G204R and N501Y along with the P80R, K417T, and E484K mutations were observed in isolates from Italy (MW642250, MW642248), the United States (MZ320527), and France (MW580244) (Multimedia Appendices 3 and 5). Conversely, the Delta variant possesses the R203M, G204R, and D377Y mutations that might cause a functional disruption in viral efficiency [14]. The trio mutations R203M, G204R, and D377Y were only observed in isolates of India (MZ702716, MZ310590, MZ310591) (Multimedia Appendices 4 and 5).

Furthermore, one of the mutations of interest in N protein was S194L, which is in a region responsible for protein oligomerization [43] (formation of hetero oligomers), and these hetero oligomers form an N-M protein complex that is critical for virus assembly [43,44]. The mutation S194L was identified with no other co-occurring mutations in isolates from India (MZ310512, MW600461-63), the United States (MW725958), and Iran (MT889692) (Multimedia Appendices 2 and 5). The S194L mutation was also identified during the SARS outbreak in 2003 [39]. In addition, another mutation, T205I, was frequently identified in the majority of the global variants evaluated, including isolates from Spain (MW715082, MW715069), France (MW580244), the United States (MW725963), and India (MW595912, MW595915, MW595914, MZ310507) (Multimedia Appendix 6).

### Mutation Hotspots in M and E Proteins of SARS-CoV-2 Variants

M protein interacts with the S and E proteins to establish the traditional shape of the virus envelope, and also helps in connecting as well as organizing other proteins of the virus [45]. We identified only five mutations in M protein in our sequence analysis: V70L (valine replaced by leucine at position 70), F28X (phenylalanine replaced by an unknown amino acid at position 28), E12X (glutamic acid replaced by an unknown amino acid at position 12), I82T (isoleucine replaced by threonine at position 82), and deletion at position 72 (Δ72) (Multimedia Appendices 7 and 8). The Δ72 deletion was observed in an isolate from Spain (MW375731), which also contains the S protein mutation D614G (Multimedia Appendix 1). The E12X and F28X mutations were observed in a UK isolate (MT906649), which also possesses the mutation D614G of S protein and the T30I and L51X mutations of E protein (Multimedia Appendix 7). The I82T mutation was present in an Indian isolate (MZ702716) that also harbored the T182I mutation of E protein (Multimedia Appendix 7); L452R, T478K, D614G, P681R mutations of S protein (Multimedia Appendix 1); and the N protein mutations R203M and D377Y from the Delta variant (Multimedia Appendix 5). The last mutation V70L was observed in an isolate from Egypt (MW533290), which stands out from all other sequences because it consists of top controversial mutations (as these mutations were present in almost every variant of SARS-CoV-2) from the S protein of the Alpha (D614G) and Delta (P681R) variants, as well as N protein mutations from the Gamma variant (R203K, G204X) (Multimedia Appendices 5, 7 and 8).

E protein of SARS-CoV-2 plays a significant role in the assembly, pathogenesis, envelope formation, and budding of the virus [7]. As the smallest of the major structural proteins, the expression of E protein is abundant inside the host cell, but only a small portion of this protein is incorporated into the virus envelope [46]. We identified five mutations in E protein: L28P (leucine replaced by proline at position 28), T30I (threonine replaced by isoleucine at position 30), L51X (leucine replaced by unknown amino acid at position 51), V58F (valine replaced by phenylalanine at position 58), and P71L (proline replaced by leucine at position 71) (Multimedia Appendices 7 and 8). The mutation V58F was present in an isolate from India (MW595915), in addition to the D614G mutation of S protein (Multimedia Appendix 1) and T205I mutation of N protein (Multimedia Appendix 5). The L28P mutation was observed only in an Iran isolate (MT994881) with no other major mutations present. The third mutation, P71L, was present in US isolates (MW725914 and MW725923), along with the D614G, R203K, and G204R mutations. The mutation P71L was also observed in an isolate from France (MW580244), along with the N501Y and E484K mutations and the A701V mutation from S protein of the Gamma variant. In contrast, mutations T30I and L51X were observed in a UK isolate (MT906649) along with D614G (from S protein), E12X, and F28X (from M protein) (Multimedia Appendices 1, 5, 7 and 8).

According to the predicted functions of these major mutations, it was concluded that four mutations from M protein and five mutations from E protein of SARS-CoV-2 variants along with other mutations of S and N proteins might increase the transmissibility, susceptibility, and lethality of the virus [8]. Additionally, analysis of the mutational patterns showed that the SARS-CoV-2 variants displayed unique mutations in isolates from different countries (Multimedia Appendix 9).

### Phylogenetic Analysis

Along with an overall visual investigation of the relevant mutations, phylogenetic analysis was performed to analyze the evolutionary relationships among different strains of SARS-CoV-2.

**Figure 1:**
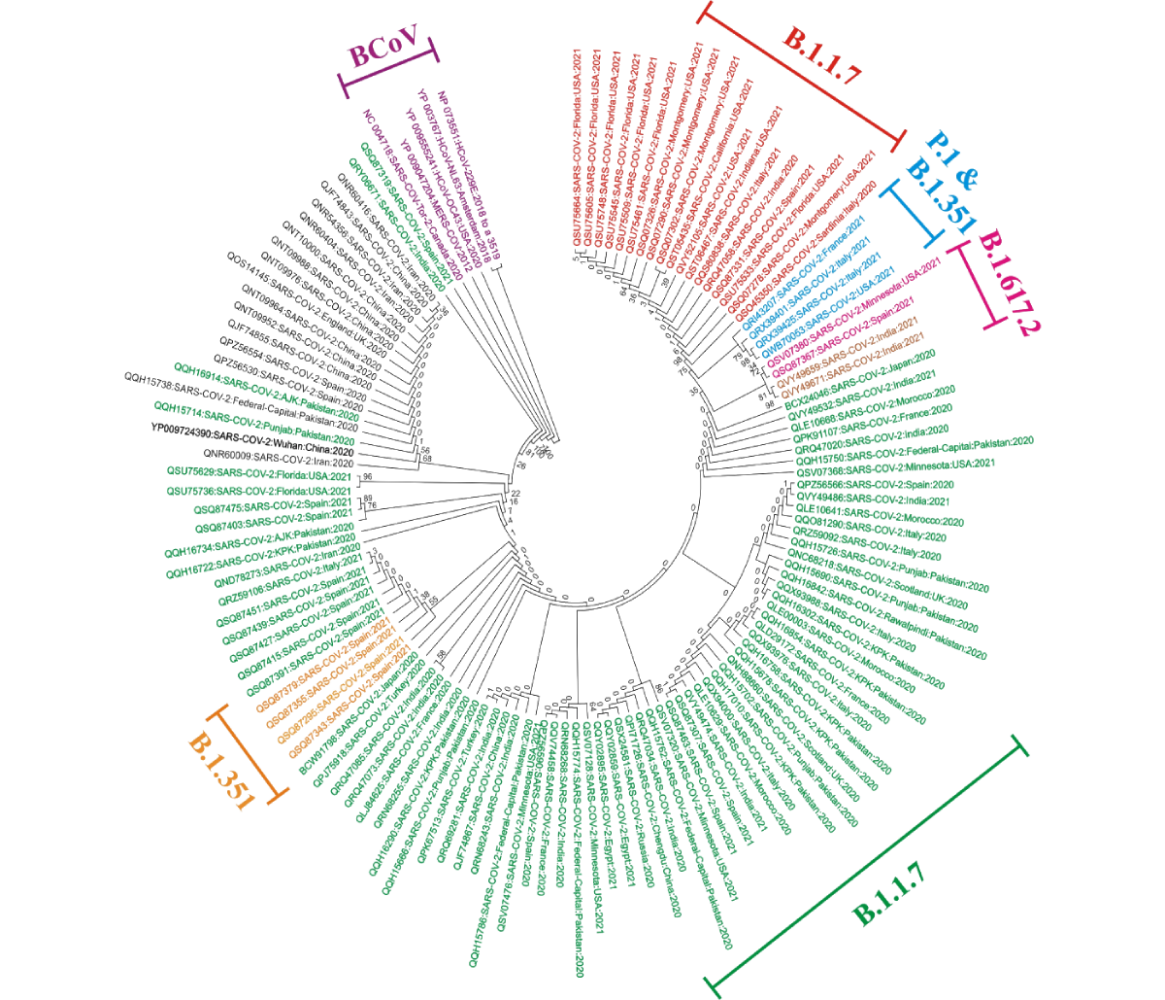
Neighbor-joining phylogenetic tree of SARS-CoV-2 spike (S) protein. The tree is divided into seven groups: Group 1 (B.1.1.7) showed 9 mutations of the Alpha variant (A501Y, A570D, D614G, P681H, T716I, S982A, D1118H, and deletion 69-70, 144 in red); Group 2 (B.1.1.7) showed the most prominent mutation (D614G in green); Group 3 (P.1 and B.1.351) of the Beta and Gamma variants comprises two mutations (E484K and N501Y in blue); Group 4 (B.1.617.2) of the Delta variant showed two mutations (D614G and L452R in pink); Group 5 (B.1.617.2) of the Delta variant showed three mutations (D614G, P681R, and L452R in brown); Group 6 (B.1.351) of the Beta variant has three mutations (D614G, L18F, and A222V in orange); and Group 7 represents the β-coronavirus (BCOV) strains used as outgroups for data comparison.

Analysis of the nodes of the tree constructed with S protein sequences showed that hCoV-NL63 (YP_003767) and hCoV-229E (NP_073551) displayed a strong association (100%), while SARS-CoV (NC_004718) and MERS-CoV (YP_009047204) exhibited strongly associated clades (76%). Moreover, the reference sequence of S protein (YP_009724392) presented a weak association (68%) and was distantly related to S protein sequences of other β-coronaviruses. In addition, the majority of the SARS-CoV-2 variants displayed no support to the reference sequence clades with some being only distantly related. Therefore, the level of observed clades in each strain differed, providing a set of contradictory nodes during cladogram comparison among S protein variants (Figure 1).

**Figure 2:**
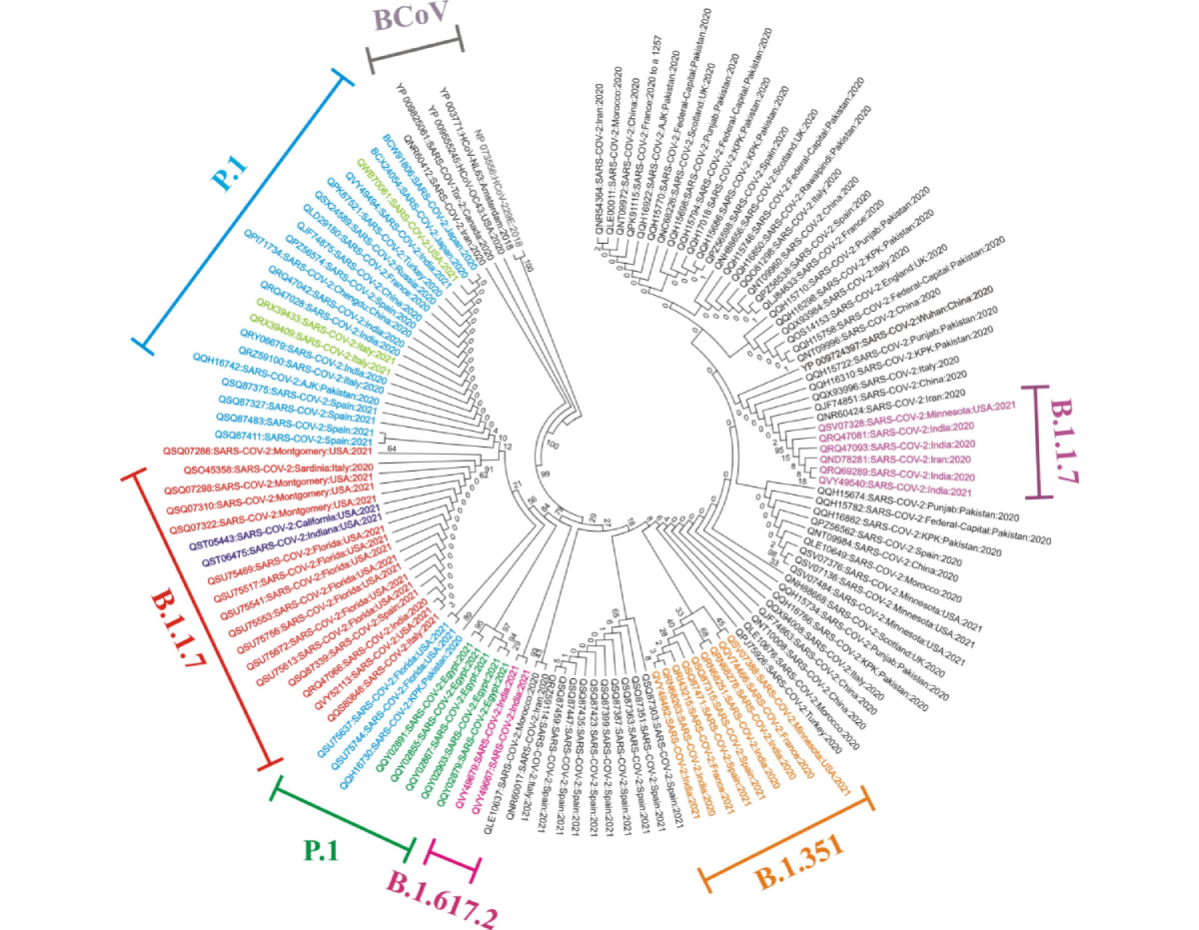
Neighbor-joining phylogenetic tree of SARS-CoV-2 nucleocapsid (N) protein. The tree is divided into eight groups: Group 1 (B.1.1.7, in red) showed four mutations of the Alpha variant (D3L, S235F, R203K, and G204R); Group 2 (B.1.1.7, in dark blue) showing three mutations (S235F, R203K, and G204R); Group 3 (B.1.1.7, in indigo) showing one mutation (S194L); Group 4 (P.1, in green) of the Gamma variant showing two mutations (R203K and G204X); Group 5 (P.1) of the Gamma variant showing two mutations (R203K and G204R, in sky blue), and P80R, R203K, G204R (in parrot green); Group 6 (B.1.351, in orange) of the Beta variant harboring the T205I mutation; Group 7 (B.1.617.2, in pink) of the Delta variant showing three mutations (D377Y, R203M, and G204R); and Group 8 representing the β-coronavirus (BCOV) strains used as outgroups for the comparison of mutations (grey).

The cladogram of N protein showed a different pattern than that constructed for S protein. All four β-coronavirus sequences of N protein exhibited strong associations among each other (100%), but there was no support for an association to the reference sequence of N protein (YP_009724393). The clades of India (QQY49667, QQY679) and the United States (QSU75744, QSU75637) were well-associated (79%-84%). The repeatability of bootstrap values below 50% was high, whereas few clades possessed weak or strong associations. Overall, no evolutionary relationship was observed among the clades of the reference sequence and retrieved nucleotide sequences (Figure 2).

The clades of M and E proteins of the examined isolates along with their reference sequences (YP_009724397 and YP_009724390) showed no association with the β-coronavirus species, whereas the β-coronavirus species displayed strong associations among themselves (100%) (Multimedia Appendix 10). Overall, the neighbor-joining trees for the four major proteins indicated total divergence among β-coronavirus species and retrieved sequences of SARS-CoV-2, and there was only weak or no support between the SARS-CoV-2 clades. The length of the branches of the neighbor-joining tree represents the genetic distance between species (Figures 1-2, Multimedia Appendix 10). Moreover, all the alternative and noncontradictory nodes as well as the repeatability of bootstrap values were rejected in this analysis.

### Conclusion

The world has witnessed a global pandemic during the 21st century and the majority of nations have contributed to the development of vaccines. Nevertheless, there have been obstacles in the distribution of the vaccines, including a lack of fundamental resources, poor immunization, and manual vaccine replication. Overall, this study can offer a better understanding of the main VoCs of SARS-CoV-2. Several new mutations were detected in this study (see Multimedia Appendix 11), which may contribute to gaining a better understanding of the VoCs as well as in identifying suitable mutations for vaccine targets. These data can further provide evidence for new types of mutations in VoCs, which will help in gaining a better understanding of the epidemiology of SARS-CoV-2 and its dynamic mutational patterns.

## Appendix

Multimedia Appendix 1 Mutations in the S protein of SARS-CoV-2 variants.

Multimedia Appendix 2 Isolates of SARS-CoV-2 Alpha variant from different countries and their mutations on S and N proteins.

Multimedia Appendix 3 Nonsynonymous mutations observed on S and N proteins of the SARS-CoV-2 Gamma variant.

Multimedia Appendix 4 Mutations in S and N proteins of SARS-CoV-2 Delta variant.

Multimedia Appendix 5 Mutations in the N protein of SARS-CoV-2 variants.

Multimedia Appendix 6 Mutations identified on S and N proteins of the Beta variant.

Multimedia Appendix 7 Mutations in the M and E proteins of SARS-CoV-2 variants.

Multimedia Appendix 8 Mutations identified from M and E proteins of SARS-CoV-2 variants.

Multimedia Appendix 9 Shift in emergence of SARS-CoV-2 variants worldwide.

Multimedia Appendix 10 Neighbor-joining phylogenetic trees of M and E proteins of SARS-CoV-2.

Multimedia Appendix 11 Novel mutations in S and N proteins of SARS-CoV-2.

## Abbreviations

ACE2: angiotensin-converting enzyme 2
E protein: envelope protein
hCoV: human coronavirus
M protein: membrane glycoprotein
MERS: Middle East Respiratory Syndrome
NCBI: National Center for Biotechnology Information
N protein: nucleocapsid protein
ORF: open reading frame
RBD: receptor-binding domain
S protein: surface glycoprotein (spike protein)
VoC: variant of concern
VoI: variant of interest

## Data Availability

The data sets generated during this study are available in the National Center for Biotechnology Information SARS-COV-2 resources repository [22].

## References

[1] Dougherty K, Mannell M, Naqvi O, Matson D, Stone J (2021). SARS-CoV-2 B.1.617.2 (Delta) variant COVID-19 outbreak associated with a gymnastics facility - Oklahoma, April-May 2021. MMWR Morb Mortal Wkly Rep.

[2] Aleem A, Samad ABA, Vaqar S (2023). Emerging variants of SARS-CoV-2 and novel therapeutics against coronavirus (COVID-19).

[3] Galloway SE, Paul P, MacCannell DR, Johansson MA, Brooks JT, MacNeil A, Slayton RB, Tong S, Silk BJ, Armstrong GL, Biggerstaff M, Dugan VG (2021). Emergence of SARS-CoV-2 B.1.1.7 lineage - United States, December 29, 2020-January 12, 2021. MMWR Morb Mortal Wkly Rep.

[4] Volz E, Mishra S, Chand M, Barrett JC, Johnson R, Geidelberg L, Hinsley WR, Laydon DJ, Dabrera G, O'Toole Á, Amato R, Ragonnet-Cronin M, Harrison I, Jackson B, Ariani CV, Boyd O, Loman NJ, McCrone JT, Gonçalves S, Jorgensen D, Myers R, Hill V, Jackson DK, Gaythorpe K, Groves N, Sillitoe J, Kwiatkowski DP, Flaxman S, Ratmann O, Bhatt S, Hopkins S, Gandy A, Rambaut A, Ferguson NM, COVID-19 Genomics UK (COG-UK) consortium (2021). Assessing transmissibility of SARS-CoV-2 lineage B.1.1.7 in England. Nature.

[5] Yang T, Yu P, Chang Y, Liang K, Tso H, Ho M, Chen W, Lin H, Wu H, Hsu SD (2021). Effect of SARS-CoV-2 B.1.1.7 mutations on spike protein structure and function. Nat Struct Mol Biol.

[6] Janik E, Niemcewicz M, Podogrocki M, Majsterek I, Bijak M (2021). The emerging concern and interest SARS-CoV-2 variants. Pathogens.

[7] Meng Bo, Kemp SA, Papa G, Datir R, Ferreira IATM, Marelli S, Harvey WT, Lytras S, Mohamed A, Gallo G, Thakur N, Collier DA, Mlcochova P, Duncan LM, Carabelli AM, Kenyon JC, Lever AM, De Marco A, Saliba C, Culap K, Cameroni E, Matheson NJ, Piccoli L, Corti D, James LC, Robertson DL, Bailey D, Gupta RK, COVID-19 Genomics UK (COG-UK) Consortium (2021). Recurrent emergence of SARS-CoV-2 spike deletion H69/V70 and its role in the Alpha variant B.1.1.7. Cell Rep.

[8] Rahman MS, Islam MR, Alam ASMRU, Islam I, Hoque MN, Akter S, Rahaman MM, Sultana M, Hossain MA (2021). Evolutionary dynamics of SARS-CoV-2 nucleocapsid protein and its consequences. J Med Virol.

[9] Tegally H, Wilkinson E, Giovanetti M, Iranzadeh A, Fonseca V, Giandhari J, Doolabh D, Pillay S, San EJ, Msomi N, Mlisana K, von Gottberg A, Walaza S, Allam M, Ismail A, Mohale T, Glass AJ, Engelbrecht S, Van Zyl G, Preiser W, Petruccione F, Sigal A, Hardie D, Marais G, Hsiao N, Korsman S, Davies M, Tyers L, Mudau I, York D, Maslo C, Goedhals D, Abrahams S, Laguda-Akingba O, Alisoltani-Dehkordi A, Godzik A, Wibmer CK, Sewell BT, Lourenço J, Alcantara LCJ, Kosakovsky Pond SL, Weaver S, Martin D, Lessells RJ, Bhiman JN, Williamson C, de Oliveira T (2021). Detection of a SARS-CoV-2 variant of concern in South Africa. Nature.

[10] Mwenda M, Saasa N, Sinyange N, Busby G, Chipimo PJ, Hendry J, Kapona O, Yingst S, Hines JZ, Minchella P, Simulundu E, Changula K, Nalubamba KS, Sawa H, Kajihara M, Yamagishi J, Kapin'a M, Kapata N, Fwoloshi S, Zulu P, Mulenga LB, Agolory S, Mukonka V, Bridges DJ (2021). Detection of B.1.351 SARS-CoV-2 variant strain - Zambia, December 2020. MMWR Morb Mortal Wkly Rep.

[11] Wu K, Werner AP, Moliva JI, Koch M, Choi A, Stewart-Jones GBE, Bennett H, Boyoglu-Barnum S, Shi W, Graham BS, Carfi A, Corbett KS, Seder RA, Edwards DK (2021). mRNA-1273 vaccine induces neutralizing antibodies against spike mutants from global SARS-CoV-2 variants. bioRxiv.

[12] Faria NR, Mellan TA, Whittaker C, Claro IM, Candido DDS, Mishra S, Crispim MAE, Sales FCS, Hawryluk I, McCrone JT, Hulswit RJG, Franco LAM, Ramundo MS, de Jesus JG, Andrade PS, Coletti TM, Ferreira GM, Silva CAM, Manuli ER, Pereira RHM, Peixoto PS, Kraemer MUG, Gaburo N, Camilo CDC, Hoeltgebaum H, Souza WM, Rocha EC, de Souza LM, de Pinho MC, Araujo LJT, Malta FSV, de Lima AB, Silva JDP, Zauli DAG, Ferreira ACDS, Schnekenberg RP, Laydon DJ, Walker PGT, Schlüter HM, Dos Santos ALP, Vidal MS, Del Caro VS, Filho RMF, Dos Santos HM, Aguiar RS, Proença-Modena JL, Nelson B, Hay JA, Monod M, Miscouridou X, Coupland H, Sonabend R, Vollmer M, Gandy A, Prete CA, Nascimento VH, Suchard MA, Bowden TA, Pond SLK, Wu C, Ratmann O, Ferguson NM, Dye C, Loman NJ, Lemey P, Rambaut A, Fraiji NA, Carvalho MDPSS, Pybus OG, Flaxman S, Bhatt S, Sabino EC (2021). Genomics and epidemiology of the P.1 SARS-CoV-2 lineage in Manaus, Brazil. Science.

[13] Washington NL, Gangavarapu K, Zeller M, Bolze A, Cirulli ET, Schiabor Barrett KM, Larsen BB, Anderson C, White S, Cassens T, Jacobs S, Levan G, Nguyen J, Ramirez JM, Rivera-Garcia C, Sandoval E, Wang X, Wong D, Spencer E, Robles-Sikisaka R, Kurzban E, Hughes LD, Deng X, Wang C, Servellita V, Valentine H, De Hoff P, Seaver P, Sathe S, Gietzen K, Sickler B, Antico J, Hoon K, Liu J, Harding A, Bakhtar O, Basler T, Austin B, MacCannell D, Isaksson M, Febbo PG, Becker D, Laurent M, McDonald E, Yeo GW, Knight R, Laurent LC, de Feo E, Worobey M, Chiu CY, Suchard MA, Lu JT, Lee W, Andersen KG (2021). Emergence and rapid transmission of SARS-CoV-2 B.1.1.7 in the United States. Cell.

[14] Zhu Z, Liu G, Meng K, Yang L, Liu D, Meng G (2021). Rapid spread of mutant alleles in worldwide SARS-CoV-2 strains revealed by genome-wide single nucleotide polymorphism and variation analysis. Genome Biol Evol.

[15] Shiehzadegan S, Alaghemand N, Fox M, Venketaraman V (2021). Analysis of the Delta variant B.1.617.2 COVID-19. Clin Pract.

[16] Cherian S, Potdar V, Jadhav S, Yadav P, Gupta N, Das M, Rakshit P, Singh S, Abraham P, Panda S, Team N (2021). SARS-CoV-2 spike mutations, L452R, T478K, E484Q and P681R, in the second wave of COVID-19 in Maharashtra, India. Microorganisms.

[17] Suratekar R, Ghosh P, Niesen MJM, Donadio G, Anand P, Soundararajan V, Venkatakrishnan AJ (2022). High diversity in Delta variant across countries revealed by genome-wide analysis of SARS-CoV-2 beyond the Spike protein. Mol Syst Biol.

[18] Syed AM, Taha TY, Tabata T, Chen IP, Ciling A, Khalid MM, Sreekumar B, Chen P, Hayashi JM, Soczek KM, Ott M, Doudna JA (2021). Rapid assessment of SARS-CoV-2-evolved variants using virus-like particles. Science.

[19] Jackson CB, Farzan M, Chen B, Choe H (2022). Mechanisms of SARS-CoV-2 entry into cells. Nat Rev Mol Cell Biol.

[20] Tamura K, Stecher G, Peterson D, Filipski A, Kumar S (2013). MEGA6: Molecular Evolutionary Genetics Analysis version 6.0. Mol Biol Evol.

[21] Chi X, Yan R, Zhang J, Zhang G, Zhang Y, Hao M, Zhang Z, Fan P, Dong Y, Yang Y, Chen Z, Guo Y, Zhang J, Li Y, Song X, Chen Y, Xia L, Fu L, Hou L, Xu J, Yu C, Li J, Zhou Q, Chen W (2020). A neutralizing human antibody binds to the N-terminal domain of the Spike protein of SARS-CoV-2. Science.

[22] SARS-CoV-2 Data. National Library of Medicine.

[23] Corpet F (1988). Multiple sequence alignment with hierarchical clustering. Nucleic Acids Res.

[24] Zhang L, Jackson CB, Mou H, Ojha A, Peng H, Quinlan BD, Rangarajan ES, Pan A, Vanderheiden A, Suthar MS, Li W, Izard T, Rader C, Farzan M, Choe H (2020). SARS-CoV-2 spike-protein D614G mutation increases virion spike density and infectivity. Nat Commun.

[25] Zhou P, Yang X, Wang X, Hu B, Zhang L, Zhang W, Si H, Zhu Y, Li B, Huang C, Chen H, Chen J, Luo Y, Guo H, Jiang R, Liu M, Chen Y, Shen X, Wang X, Zheng X, Zhao K, Chen Q, Deng F, Liu L, Yan B, Zhan F, Wang Y, Xiao G, Shi Z (2020). A pneumonia outbreak associated with a new coronavirus of probable bat origin. Nature.

[26] Ostrov DA (2021). Structural consequences of variation in SARS-CoV-2 B.1.1.7. J Cell Immunol.

[27] Liu H, Yuan M, Huang D, Bangaru S, Zhao F, Lee CD, Peng L, Barman S, Zhu X, Nemazee D, Burton DR, van Gils MJ, Sanders RW, Kornau H, Reincke SM, Prüss H, Kreye J, Wu NC, Ward AB, Wilson IA (2021). A combination of cross-neutralizing antibodies synergizes to prevent SARS-CoV-2 and SARS-CoV pseudovirus infection. Cell Host Microbe.

[28] Liu Y, Liu J, Plante KS, Plante JA, Xie X, Zhang X, Ku Z, An Z, Scharton D, Schindewolf C, Widen SG, Menachery VD, Shi PY, Weaver SC (2022). The N501Y spike substitution enhances SARS-CoV-2 infection and transmission. Nature.

[29] McCarthy KR, Rennick LJ, Nambulli S, Robinson-McCarthy LR, Bain WG, Haidar G, Duprex WP (2021). Recurrent deletions in the SARS-CoV-2 spike glycoprotein drive antibody escape. Science.

[30] La Rosa G, Mancini P, Bonanno Ferraro G, Veneri C, Iaconelli M, Lucentini L, Bonadonna L, Brusaferro S, Brandtner D, Fasanella A, Pace L, Parisi A, Galante D, Suffredini E (2021). Rapid screening for SARS-CoV-2 variants of concern in clinical and environmental samples using nested RT-PCR assays targeting key mutations of the spike protein. Water Res.

[31] Patro LPP, Sathyaseelan C, Uttamrao PP, Rathinavelan T (2021). The evolving proteome of SARS-CoV-2 predominantly uses mutation combination strategy for survival. Comput Struct Biotechnol J.

[32] Wise J (2021). Covid-19: The E484K mutation and the risks it poses. BMJ.

[33] Starr TN, Greaney AJ, Hilton SK, Ellis D, Crawford KHD, Dingens AS, Navarro MJ, Bowen JE, Tortorici MA, Walls AC, King NP, Veesler D, Bloom JD (2020). Deep mutational scanning of SARS-CoV-2 receptor binding domain reveals constraints on folding and ACE2 binding. Cell.

[34] Hodcroft EB, Zuber M, Nadeau S, Vaughan TG, Crawford KHD, Althaus CL, Reichmuth ML, Bowen JE, Walls AC, Corti D, Bloom JD, Veesler D, Mateo D, Hernando A, Comas I, González Candelas F, Stadler T, Neher RA, SeqCOVID-SPAIN Consortium (2021). Emergence and spread of a SARS-CoV-2 variant through Europe in the summer of 2020. medRxiv.

[35] Nonaka CKV, Franco MM, Gräf T, de Lorenzo Barcia CA, de Ávila Mendonça RN, de Sousa KAF, Neiva LMC, Fosenca V, Mendes AVA, de Aguiar RS, Giovanetti M, de Freitas Souza BS (2021). Genomic evidence of SARS-CoV-2 reinfection involving E484K spike mutation, Brazil. Emerg Infect Dis.

[36] Peacock TP, Sheppard CM, Brown JC, Goonawardane N, Zhou J, Whiteley M, de Silva TI, Barclay WS, PHE Virology Consortium (2021). The SARS-CoV-2 variants associated with infections in India, B. 1.617, show enhanced spike cleavage by furin. BioRxiv.

[37] Zhao S, Lou J, Chong MKC, Cao L, Zheng H, Chen Z, Chan RWY, Zee BCY, Chan PKS, Wang MH (2021). Inferring the association between the risk of COVID-19 case fatality and N501Y substitution in SARS-CoV-2. Viruses.

[38] Plante JA, Liu Y, Liu J, Xia H, Johnson BA, Lokugamage KG, Zhang X, Muruato AE, Zou J, Fontes-Garfias CR, Mirchandani D, Scharton D, Bilello JP, Ku Z, An Z, Kalveram B, Freiberg AN, Menachery VD, Xie X, Plante KS, Weaver SC, Shi P (2021). Spike mutation D614G alters SARS-CoV-2 fitness. Nature.

[39] Wu S, Tian C, Liu P, Guo D, Zheng W, Huang X, Zhang Y, Liu L (2021). Effects of SARS-CoV-2 mutations on protein structures and intraviral protein-protein interactions. J Med Virol.

[40] Zhou P, Shi Z (2021). SARS-CoV-2 spillover events. Science.

[41] Funk T, Pharris A, Spiteri G, Bundle N, Melidou A, Carr M, Gonzalez G, Garcia-Leon A, Crispie F, O'Connor L, Murphy N, Mossong J, Vergison A, Wienecke-Baldacchino AK, Abdelrahman T, Riccardo F, Stefanelli P, Di Martino A, Bella A, Lo Presti A, Casaca P, Moreno J, Borges V, Isidro J, Ferreira R, Gomes JP, Dotsenko L, Suija H, Epstein J, Sadikova O, Sepp H, Ikonen N, Savolainen-Kopra C, Blomqvist S, Möttönen T, Helve O, Gomes-Dias J, Adlhoch C, COVID study groups (2021). Characteristics of SARS-CoV-2 variants of concern B.1.1.7, B.1.351 or P.1: data from seven EU/EEA countries, weeks 38/2020 to 10/2021. Euro Surveill.

[42] Martins AF, Zavascki AP, Wink PL, Volpato FCZ, Monteiro FL, Rosset C, De-Paris F, Barth AL, Ramos (2021). Detection of SARS-CoV-2 lineage P.1 in patients from a region with exponentially increasing hospitalisation rate, February 2021, Rio Grande do Sul, Southern Brazil. Euro Surveill.

[43] Yu I, Oldham ML, Zhang J, Chen J (2006). Crystal structure of the severe acute respiratory syndrome (SARS) coronavirus nucleocapsid protein dimerization domain reveals evolutionary linkage between corona- and arteriviridae. J Biol Chem.

[44] He R, Leeson A, Ballantine M, Andonov A, Baker L, Dobie F, Li Y, Bastien N, Feldmann H, Strocher U, Theriault S, Cutts T, Cao J, Booth TF, Plummer FA, Tyler S, Li X (2004). Characterization of protein-protein interactions between the nucleocapsid protein and membrane protein of the SARS coronavirus. Virus Res.

[45] Siu YL, Teoh KT, Lo J, Chan CM, Kien F, Escriou N, Tsao SW, Nicholls JM, Altmeyer R, Peiris JSM, Bruzzone R, Nal B (2008). The M, E, and N structural proteins of the severe acute respiratory syndrome coronavirus are required for efficient assembly, trafficking, and release of virus-like particles. J Virol.

[46] Schoeman D, Fielding BC (2019). Coronavirus envelope protein: current knowledge. Virol J.

