# Correction: Mutations of SARS-CoV-2 Structural Proteins in the Alpha, Beta, Gamma, and Delta Variants: Bioinformatics Analysis

**DOI:** 10.2196/64915

**Published:** 2024-08-05

**Authors:** Saima Rehman Khetran, Roma Mustafa

**Affiliations:** 1 Department of Life Sciences Sardar Bahadur Khan Women's University Quetta Pakistan

In “Mutations of SARS-CoV-2 Structural Proteins in the Alpha, Beta, Gamma, and Delta Variants: Bioinformatics Analysis” (JMIR Bioinform Biotech 2023;4:e43906) the authors made one addition.

An additional citation [31] was added to the Results and Discussion Section, which previously appeared as:

Apart from these mutations, deletions at position 85-89 (Δ85-Δ89) in a Spanish isolate (MW715071) along with other unique mutations of S protein, such as V90T (in which valine is replaced by threonine at position 90), A93Y (in which alanine is replaced by tyrosine at position 93), and D138H (in which aspartic acid is replaced by histidine at position 138), were also observed (Multimedia Appendices 1 and 2).

This has been changed as follows:

Apart from these mutations, deletions at position 85-89 (Δ85-Δ89) in a Spanish isolate (MW715071) along with other unique mutations of S protein, such as V90T (in which valine is replaced by threonine at position 90) [31], A93Y (in which alanine is replaced by tyrosine at position 93), and D138H (in which aspartic acid is replaced by histidine at position 138), were also observed (Multimedia Appendices 1 and 2).

The reference being included will be added to the References section, resulting in the renumeration of all references following Reference 31. The reference being added is the following:

31. Stojanov D. Phylogenicity of B.1.1.7 surface glycoprotein, novel distance function and first report of V90T missense mutation in SARS-CoV-2 surface glycoprotein. Meta Gene. 2021;30:100967. doi:https://doi.org/10.1016/j.mgene.2021.100967

The correction will appear in the online version of the paper on the JMIR Publications website on August 5, 2024, together with the publication of this correction notice. Because this was made after submission to PubMed, PubMed Central, and other full-text repositories, the corrected article has also been resubmitted to those repositories.

