# Supplementary material for: Mutations of SARS-CoV-2 Structural Proteins in the Alpha, Beta, Gamma, and Delta Variants: Bioinformatics Analysis
Source: JMIR Bioinform Biotechnol. 2023 Jul 14;4:e43906. doi: 10.2196/43906 (PMC10353769; doi:10.2196/43906)
Supplement: Multimedia Appendix 1 [file bioinform_v4i1e43906_app1.docx]

**(A)**

**
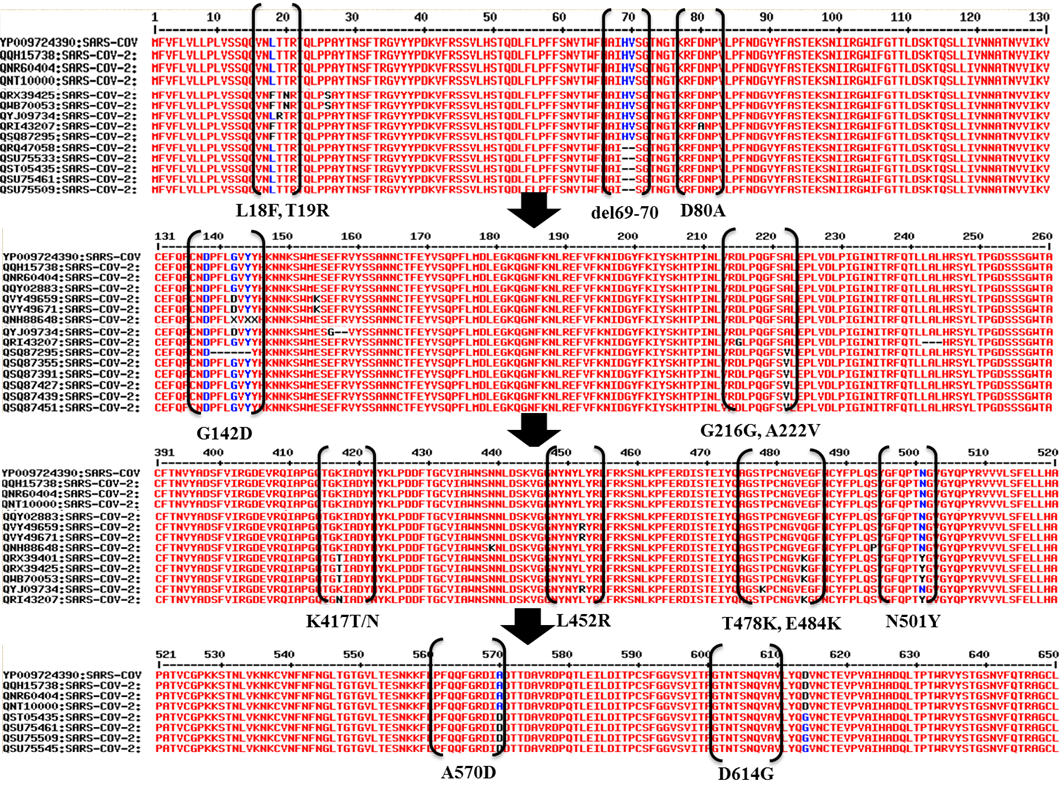
**

**(B)**

**
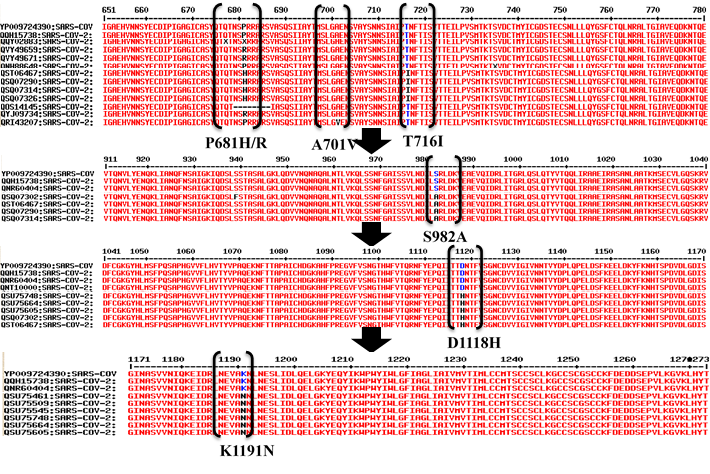
**

**(A)** Mutations in the S protein of SARS-COV-2 variants (aa1-aa650): first row consists of 3 mutations L18F, T19R, D80A and one deletion at 69-70position. Second row consists of three mutations: G142D, G216G and A222V. Third row has 5 mutations K417T/N, L452R, T478K, F484K and N501Y. Last row contains 2 mutations only A570D and D614G. Normal amino acid sequences are highlighted (red and blue) and mutations in amino acids are shown by black color. **(B)** Mutations in the S protein of SARS-COV-2 variants from amino acid aa651-aa1273: first row consists of 3 mutations P681H/R, A701V, and T716I. Second row consists of single mutation S982A. Third row also possess single mutation D1118H. Last row has single mutation K1191N only. Normal amino acid sequences highlighted in red and blue while mutations in amino acids are shown by black color.
