# Supplementary material for: Mutations of SARS-CoV-2 Structural Proteins in the Alpha, Beta, Gamma, and Delta Variants: Bioinformatics Analysis
Source: JMIR Bioinform Biotechnol. 2023 Jul 14;4:e43906. doi: 10.2196/43906 (PMC10353769; doi:10.2196/43906)
Supplement: Multimedia Appendix 2 [file bioinform_v4i1e43906_app2.docx]

Isolates of Alpha variant from different countries and their mutations present on S and N protein of SARS-CoV-2

| **UK VARIANT 20I/N501Y.V1 (LINEAGE B.1.1.7)** | | | | | | | | | | |
| --- | --- | --- | --- | --- | --- | --- | --- | --- | --- | --- |
| **SURFACE GLYCO PROTEIN** | | | | | | **NUCLEOCAPSID PHOSPHOPROTEIN** | | | | |
| **Accession Number** | **PROTEIN ID** | **States** | **Non-synonymous muta**  **tion** | **Deletion** | **Novel mutations** | **Accession Number** | **PROTEIN ID** | **States** | **Non-synony-**  **mous mutation** | **Novel mutations** |
| **MW642250**  **MW642248** | QRX39425  QRX39401 | Italy  Italy | N501Y  (protein oligero  merization) |  |  | **MW422034** | QQH16298 | Pakistan |  | L139F |
| **MW711159**  **MZ311101**  **MW725906**  **MW600456**  **MW491232** | QSO45350  QVY52105  QSU75533  QRQ47058  QQS80838 | Italy  USA  USA  India  Italy | N501Y  A570D  D614G  P681H  T716I  S982A  D1118H  (increase in virus lethality and transmissibility) | 69-70  144 |  | **MW711159**  **MW712862**  **MW712863**  **MW712864**  **MZ311101**  **MW725900**  **MW725904**  **MW712861** | QSO45358  QSQ07298  QSQ07310  QSQ07322  QVY52113  QSU75469  QSU75517  QSQ07286 | Italy  USA  USA  USA  USA  USA  USA  USA | D3L  S235F  R203K  G204R  (change in protein structure morphology) |  |
| **MW712861** | QSQ07278 | USA | N501Y  A570D  P681H  T716I  S982A  D1118H | 69-70  144 |  | **MW715071** | QSQ87339 | Spain | D3L  R203K  G204R  S235F | 209-11 (deletion)  G212S |
| **MW725912**  **MW725900**  **MW725904**  **MW725907**  **MW725924**  **MW725917**  **MW712862**  **MW712864**  **MW712865** | QSU75605  QSU75461  QSU75509  QSU75545  QSU75748  QSU75664  QSQ07290  QSQ07314  QSQ07326 | USA  USA  USA  USA  USA  USA  USA  USA  USA | N501Y  A570D  D614G  P681H  T716I  S982A  D1118H  K1191N | 69-70  144 | G72W | **MW725912**  **MW491232**  **MW600456**  **MW725906**  **MW725907**  **MW725924**  **MW725917** | QSU75613  QQS80846  QRQ47066  QSU75541  QSU75553  QSU75756  QSU75672 | USA  Italy  India  USA  USA  USA  USA | D3L  S235F  R203K  G204R |  |
| **MW721338** | QST05435 | USA | N501Y  A570D  D614G  P681H  T716I  S982A  D1118H | 69-70  144 | L1203R | **MW721338**  **MW721424** | QST05443  QST06475 | USA  USA | R203K  G204R  S235F |  |
| **MW712863** | QSQ07302 | USA | N501Y  A570D  D614G  P681H  T716I  S982A  D1118H | 69-70  144 | S939F | **MW725958**  **MZ310512**  **MW600461**  **MW600463**  **MT889692** | QSV07328  QVY49540  QRQ47081  QRQ47093  QND78281 | USA  India  India  India  Iran | S194L (change in protein structure morphology) |  |
| **MW721424** | QST06467 | USA | N501Y  A570D  D614G  P681H  T716I  S982A  D1118H | 69-70  144 | S221L | **MW725962**  **MW725957**  **MW725971** | QSV07376  QSV07316  QSV07484 | USA  USA  USA |  | P199L  P67S |
| **MW715071** | QSQ87331 | Spain | N501Y A570D  D614G  P681H  T716I  S982A  D1118H | 69-70  144  85-89 | V90T  A93Y  D138H | **MW725923**  **MW725914** | QSU75744  QSU75637 | USA  USA | R203K  G204R | I292T |
| **MW725914**  **MW725923** | QSU75629  QSU75736 | USA  USA | D614G |  | G257S  Q414R  Q614R  T778I | **MW715068**  **MW715072**  **MW715073**  **MW715075**  **MW715080**  **MW715079**  **MW715078**  **MW715076** | QSQ87303  QSQ87351  QSQ87363  QSQ87387  QSQ87447  QSQ87435  QSQ87423  QSQ87399 | Spain  Spain  Spain  Spain  Spain  Spain  Spain  Spain | A220V |  |
| **MZ320527** | QWB70053 | USA | N501Y  D614G |  |  |  |  |  |  |  |
| **MW725958**  **MW600453**  **MW600436** | QSV07320  QRQ47034  QRQ47020 | USA  India  India | P681H  D614G  (enhanced the host fusion capability) |  |  | **MW421989** | QQH15758 | Pakistan |  | S237X |
| **MW422081** | QQH16854 | Pakistan | D614G |  | S813N | **MW715081** | QSQ87459 | Spain | A220V | H300Y |
| **MW421986** | QQH15714 | Pakistan | D614G |  | P26L | **MT994632** | QNR54364 | Iran |  | S186F |
| **MT994881** | QNR60416 | Iran |  |  | G219R | **MT994632** | QNR60017 | Iran |  | G215R |
| **MT994632** | QNR54356 | Iran |  |  | T22I | **MW375726** | QPZ56538 | Spain |  | S197L |
| **MW412338** | QJF74843 | China |  |  | V367F | **MT731292** | QLE10637 | Morocco | D348H |  |
| **S protein mutations with Mutation D614G in alpha variant** | | | | | | | | | | |
| **BS000686** | BCW91798 | Japan | D614G |  | Q613H | **MW422070** | QQH16722 | Pakistan | D614G | Q1207H |
| **MT777677** | QLJ84625 | France | D614G |  | S868G | **MW422071** | QQH16734 | Pakistan | D614G | T1117I |
| **MZ310512** | QVY49532 | India | D614G |  | P681R | **MW422035** | QQH16302 | Pakistan | D614G | D1163Y |
| **MW741552**  **MT882022**  **MT731468**  **MT731327**  **MT731292**  **MT731285**  **MW421982**  **MW421983**  **MW421984**  **MW421985**  **MW421987**  **MW421989**  **MW421990**  **MW421991**  **MW421992**  **MW422034**  **MW422073**  **MW422080**  **MW422094** | QSX24581  QNC68218  QLE10668  QLE10641  QLE10629  QLE00003  QQH15666  QQH15678  QQH15690  QQH15702  QQH15726  QQH15750  QQH15762  QQH15774  QQH15786  QQH16290  QQH16758  QQH16842  QQH17010 | Russia  UK  Morocco  Morocco  Morocco  Morocco  Pakistan  Pakistan  Pakistan  Pakistan  Pakistan  Pakistan  Pakistan  Pakistan  Pakistan  Pakistan  Pakistan  Pakistan  Pakistan | D614G |  |  | **MW320691**  **MW308549**  **BS000701**  **MT412340**  **MW301121**  **MZ310507**  **MW595914**  **MW595912**  **MW595915**  **MW375731**  **MW468415**  **MW530512**  **MW530511**  **MW530510**  **MW652721**  **MW725962**  **MW725942**  **MW725971** | QPK67513  QPJ75918  BCX24046  QJF74867  QPI71726  QVY49474  QRN68255  QRN68243  QRN68268  QPZ56590  QQO81290  QQX94000  QQX93988  QQX93976  QRZ59092  QSV07368  QSV07128  QSV07476 | Turkey  Turkey  Japan  China  China  India  India  India  India  Spain  Italy  Italy  Italy  Italy  Italy  USA  USA  USA | D614G  (higher transmissibility and lethality) |  |
| **MT906649** | QNH88648 | UK | D614G |  | T22X,  P25X  G142X  Y144X  Y145X  N440K  N439K  S494P  S735X  K1191X | **MW715073**  **MW715076**  **MW715079**  **MW715080**  **MW715081**  **MW652728** | QSQ87355  QSQ87391  QSQ87427  QSQ87439  QSQ87451  QRZ59106 | Spain  Spain  Spain  Spain  Spain  Italy | D614G | A222V |
| **MW533286**  **MW533289** | QQY02847  QQY02883 | Egypt  Egypt | D614G |  | Q23X  S12X  Q677X  P681X | **MZ310591**  **MZ310590** | QVY49671  QVY49659 | India  India | D614G | G142D  E154K  L452R  P681R  Q1071H  H1101D |
| **MW533290** | QQY02895 | Egypt | D614G |  | T859I  Q677H  P681R  (increase viral lethality) | **MW533288** | QQY02871 | Egypt | D614G | S12X  Q677X  P681X |
| **MW725963** | QSV07380 | USA | D614G |  | S13I  L452R  W152C | **MT889692** | QND78273 | Iran | D614G | H146R  N1192S  D808G |
| **MW645476** | QRY06671 | India | D614G |  | F490S  S943P | **MW533287** | QQY02859 | Egypt | D614G | Q677H  P681L |
| **MZ310508** | QVY49486 | India | D614G |  | D80Y | **MW422086** | QQH16914 | Pakistan |  | D80Y |
| **MW059036** | QOS14145 | UK |  | 679-686 |  | **MW505982** | QQV74458 | France | D614G | S477N |
| **MT906650** | QNH88660 | UK | D614G |  | N439K | **MW600654** | QRQ69281 | India | D614G | L54F |
| **MW600461**  **MW600463** | QRQ47073  QRQ47085 | India  India | D614G |  | M1050I | **MW715070** | QSQ87319 | Spain | D614G | T478K  T732A |
| **MT994632** | QNR60009 | Iran |  |  | C488R  L948I | **MW715069**  **MW715082** | QSQ87307  QSQ87463 | Spain | D614G | S12F  D627A |
| **MW715074** | QSQ87367 | Spain | D614G |  | G769V  L452R | **MW715077**  **MW715083** | QSQ87403  QSQ87475 | Spain  Spain | D614G | T20I  T1117I |
| **MT709104**  **MW322968** | QLD29172  QPK91107 | France  France | D614G |  |  | **MW375729** | QPZ56566 | Spain | D614G | G1251V |
