# Supplementary material for: Mutations of SARS-CoV-2 Structural Proteins in the Alpha, Beta, Gamma, and Delta Variants: Bioinformatics Analysis
Source: JMIR Bioinform Biotechnol. 2023 Jul 14;4:e43906. doi: 10.2196/43906 (PMC10353769; doi:10.2196/43906)
Supplement: Multimedia Appendix 3 [file bioinform_v4i1e43906_app3.docx]

Non-synonymous mutations observed on S and N proteins of the SARS-COV-2 Gamma variant

| **BRAZIL VARIANT LINEAGE P.1 GR/501Y.V3** | | | | | | | | | |
| --- | --- | --- | --- | --- | --- | --- | --- | --- | --- |
| **SURFACE GLYCO PROTEIN** | | | | | **NUCLEOCAPSID PHOSPHOPROTEIN** | | | | |
| **Accession #** | **Protein id** | **Country** | **Non-synonymous mutations** | **novel mutations** | **Accession #** | **Protein id** | **Country** | **Non-synonymous mutations** | **Novel**  **mutations** |
| **MW642250**  **MW642248**  **MZ320527** | QRX39425  QRX39401  QWB70053 | Italy  Italy  USA | N501Y  E484K  K417T | L18F,  T20N  P26S  R190S  D138Y H655Y T1027I  V1176F | **MW715074**  **MW422071**  **MW652721**  **MW645476**  **BS000701**  **MW301121**  **BS000686**  **MW375729**  **MT412340**  **MW741552**  **MW320691**  **MT709104**  **MZ310508** | QSQ87375  QQH16742  QRZ59100  QRY06679  BCX24054  QPI71734  BCW91806  QPZ56574  QJF74875  QSX24589  QPK67521  QLD29180  QVY49494 | Spain  Pakistan  Italy  India  Japan  China  Japan  Spain  China  Russia  Turkey  France  India |  |  |
| **MW580244** | QRI43207 | France | N501Y  E484K  A701V | L18F  D80A  K417N  D215G  243-245 (deletion) | **MW642248**  **MW642250**  **MZ320527** | QRX39409  QRX39433  QWB70061 | Italy  Italy  USA | P80R  R203K  G204R |  |
| **MW711159**  **MW491232** | QSO45350  QQS80838 | Italy  Italy | N501Y |  | **MW422070** | QQH16730 | Pakistan | R203K  G204R | A152X |
|  |  |  |  |  | **MW715070** | QSQ87327 | Spain | R203K  G204R | Q418H |
|  |  |  |  |  | **MW600436** | QRQ47028 | India | R203K  G204R | M210I |
|  |  |  |  |  | **MW600453** | QRQ47042 | India | G204R | M210I |
|  |  |  |  |  | **MW533286**  **MW533289** | QQY02855  QQY02891 | Egypt  Egypt | R203K  G204X | G212X  G25X |
|  |  |  |  |  | **MW533288** | QQY02879 | Egypt | R203K  G204X | G212V  G25X |
|  |  |  |  |  | **MW533287**  **MW533290** | QQY02867  QQY02903 | Egypt  Egypt | R203K  G204X | M212V |
|  |  |  |  |  | **MW715077**  **MW715083** | QSQ87411  QSQ87483 | Spain  Spain | R203K  G204R | H145Y |
