# Supplementary material for: Mutations of SARS-CoV-2 Structural Proteins in the Alpha, Beta, Gamma, and Delta Variants: Bioinformatics Analysis
Source: JMIR Bioinform Biotechnol. 2023 Jul 14;4:e43906. doi: 10.2196/43906 (PMC10353769; doi:10.2196/43906)
Supplement: Multimedia Appendix 4 [file bioinform_v4i1e43906_app4.docx]

Mutations in S and N proteins of SARS-CoV-2 Delta variant

| **INDIAN VARIANT (LINEAGE B.1.617.2) (DELTA VARIANT)** | | | | | | | | | |
| --- | --- | --- | --- | --- | --- | --- | --- | --- | --- |
| **SURFACE GLYCO PROTEIN** | | | | | **NUCLEOCAPSID PHOSPHOPROTEIN** | | | | |
| **Accession #** | **Protein id** | **Country** | **Non-synonymous mutations** | **Additional mutations** | **Accession #** | **Protein id** | **Country** | **Non-synonymous mutations** | **Additional mutations** |
| **MZ702716** | QYJ09734 | India | L452R  T478K  D614G  P681R  T19R | E156G  A1020R  156-7 deletion | **MZ702716** | QYJ09742 | India | R203M  D377Y  D63G |  |
| **MZ310590**  **MZ310591** | QVY49659  QVY49671 | India  India | G142D  L452R  P681R  D614G | E154K  Q1071H  H1101D | **MZ310590**  **MZ310591** | QVY49667  QVY49679 | India  India | R203M  G204R  D377Y |  |
| **MW533290** | QQY02895 | Egypt | P681R  D614G | T859I  Q677H |  |  |  |  |  |
| **MW725963** | QSV07380 | USA | D614G  L452R | S13I  W152C |  |  |  |  |  |
| **MW715074** | QSQ87367 | Spain | D614G  L452R | G769V |  |  |  |  |  |
| **MW715070** | QSQ87319 | Spain | D614G  T478K | T732A |  |  |  |  |  |
