# Supplementary material for: Mutations of SARS-CoV-2 Structural Proteins in the Alpha, Beta, Gamma, and Delta Variants: Bioinformatics Analysis
Source: JMIR Bioinform Biotechnol. 2023 Jul 14;4:e43906. doi: 10.2196/43906 (PMC10353769; doi:10.2196/43906)
Supplement: Multimedia Appendix 5 [file bioinform_v4i1e43906_app5.docx]

**(A)**

**
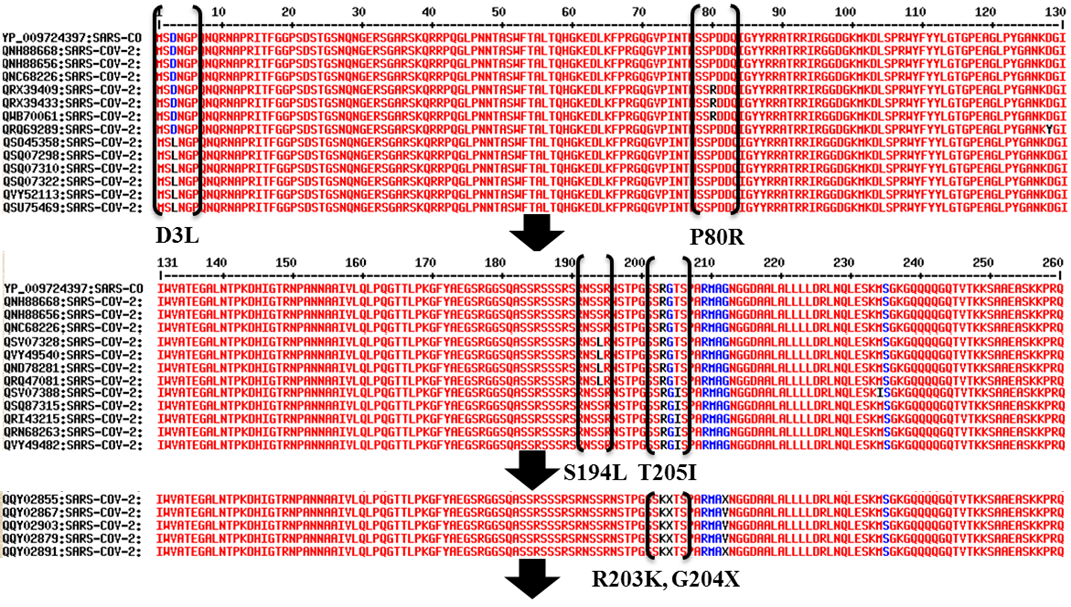
**

**(B)**

**
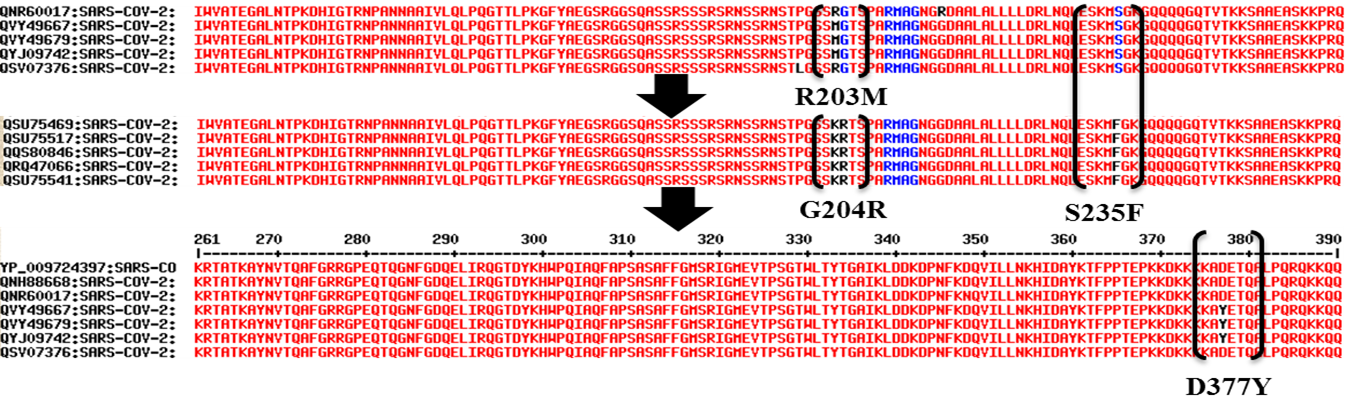
**

**(A)** Mutations in the N protein of SARS-COV-2 variants (aa1-aa260): first row consists of 2 mutations D3L and P80R. Second row contains two mutations S194L and T205I. Third row has 2 mutations R203K and G204R. Normal amino acid sequences highlighted in red and blue while mutations in amino acids are shown by black color. **(B)** Mutations in the N protein of SARS-COV-2 variants: first andsecond row ranges from amino acid aa131-aa260 consists of 3 mutations R203M, S235F and G204R. Last row from aa261-aa390 consists of single mutation D377Y. normal aa-substitutions highlighted in blue and red while mutations are highlighted in black color.
