# Supplementary material for: Mutations of SARS-CoV-2 Structural Proteins in the Alpha, Beta, Gamma, and Delta Variants: Bioinformatics Analysis
Source: JMIR Bioinform Biotechnol. 2023 Jul 14;4:e43906. doi: 10.2196/43906 (PMC10353769; doi:10.2196/43906)
Supplement: Multimedia Appendix 6 [file bioinform_v4i1e43906_app6.docx]

Mutations identified on S and N proteins of the Beta variant

| **BETA VARIANT 20J/501Y.V2 LINEAGE B.1.351 (SOUTH AFRICA)** | | | | | | | | | |
| --- | --- | --- | --- | --- | --- | --- | --- | --- | --- |
| **SURFACE GLYCO PROTEIN** | | | | | **NUCLEOCAPSID PHOSPHOPROTEIN** | | | | |
| **Accession #** | **Protein id** | **Country** | **Non-synonymous mutations** | **novel mutations** | **Accession #** | **Protein id** | **Country** | **Non-synonymous mutations** | **Novel mutations** |
| **MW580244** | QRI43207 | France | N501Y  E484K  A701V  L18F  D80A  K417N  D215G | T20N  P26S  R190S D138Y H655Y T1027I  V1176F | **MW715082**  **MZ310507**  **MW595914**  **MW580244**  **MW715069** | QSQ87471  QVY49482  QRN68263  QRI43215  QSQ87315 | Spain  India  India  France  Spain | T205I |  |
| **MW642250**  **MW642248**  **MZ320527** | QRX39425  QRX39401  QWB70053 | Italy  Italy  USA | L18F,  N501Y  E484K  K417T | 243-245 (deletion) | **MW595912**  **MW595915** | QRN68251  QRN68276 | India  India | T205I | M317I |
| **MW715072** | QSQ87343 | Spain | D614G  L18F A222V | K1204R | **MW725963** | QSV07388 | USA | T205I | M234I |
| **MW715075** | QSQ87379 | Spain | D614G  L18F  A222V |  | **MW505982** | QQV74466 | France |  | A376T  M234I |
| **MW715068**  **MW715078** | QSQ87295  QSQ87415 | Spain  Spain | L18F  A222V  D614G | Deletion  139-144 |  |  |  |  |  |
