# Supplementary material for: Mutations of SARS-CoV-2 Structural Proteins in the Alpha, Beta, Gamma, and Delta Variants: Bioinformatics Analysis
Source: JMIR Bioinform Biotechnol. 2023 Jul 14;4:e43906. doi: 10.2196/43906 (PMC10353769; doi:10.2196/43906)
Supplement: Multimedia Appendix 7 [file bioinform_v4i1e43906_app7.docx]

**
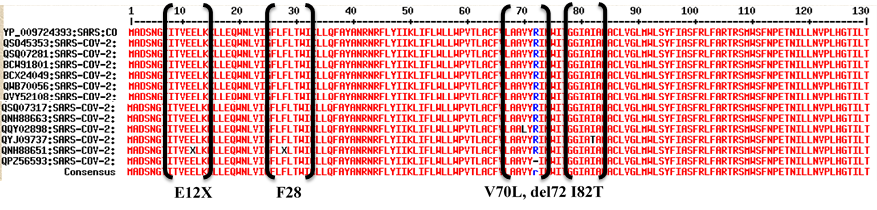
**

Mutations in the M protein of SARS-COV-2 variants (aa1-aa130): it includes a total of 4 mutations E12X, F28X, V70L, I82T and 1 deletion at 72position. Normal aa-substitutions highlighted in blue and red while mutations are highlighted in black color.

**
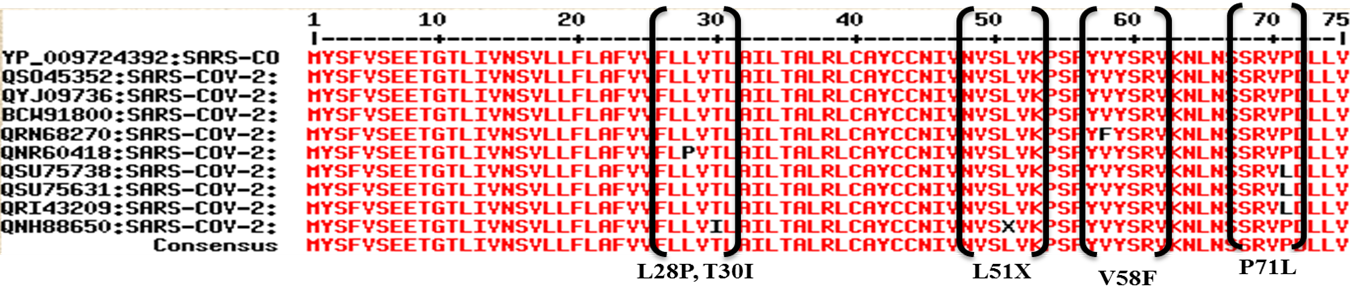
**

Mutations in the E protein of SARS-COV-2 variants (aa1-aa75): it includes a total of 5 mutations L28P, T30I, L51X, V58F and P71L. Normal aa-substitutions highlighted in blue and red while mutations are highlighted in black color.
