# Supplementary material for: Mutations of SARS-CoV-2 Structural Proteins in the Alpha, Beta, Gamma, and Delta Variants: Bioinformatics Analysis
Source: JMIR Bioinform Biotechnol. 2023 Jul 14;4:e43906. doi: 10.2196/43906 (PMC10353769; doi:10.2196/43906)
Supplement: Multimedia Appendix 8 [file bioinform_v4i1e43906_app8.docx]

Mutations identified from M and E proteins of SARS-CoV-2 variants

| **MEMBRANE GLYCOPROTEIN** | | | | **ENVELOPE PROTEIN** | | | |
| --- | --- | --- | --- | --- | --- | --- | --- |
| **Accession #** | **Protein id** | **Country** | **Mutations** | **Accession #** | **Protein id** | **Country** | **Mutation** |
| **MW375731** | QPZ56593 | Spain | 72 (deletion) | **MW595915** | QRN68270 | India | V58F |
| **MZ702716** | QYJ09737 | India | I82T | **MT994881** | QNR60418 | Iran | L28P |
| **MT906649** | QNH88651 | UK | E12X  F28X | **MT906649** | QNH88650 | UK | T30I  L51X |
| **MW533290** | QQY02898 | Egypt | V70L | **MW580244**  **MW725914**  **MW725923** | QRI43209  QSU75631  QSU75738 | France  USA  USA | P71L |
