# Supplementary material for: Mutations of SARS-CoV-2 Structural Proteins in the Alpha, Beta, Gamma, and Delta Variants: Bioinformatics Analysis
Source: JMIR Bioinform Biotechnol. 2023 Jul 14;4:e43906. doi: 10.2196/43906 (PMC10353769; doi:10.2196/43906)
Supplement: Multimedia Appendix 9 [file bioinform_v4i1e43906_app9.docx]

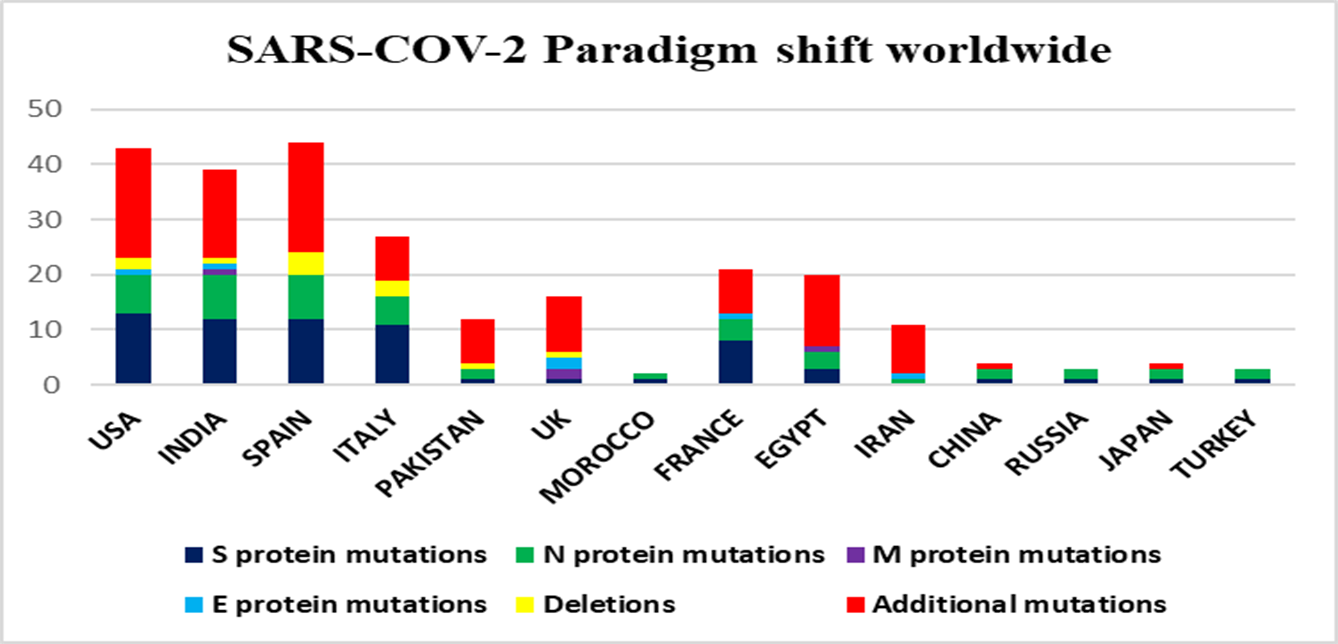


Shift in emergence of SARS-CoV-2 variants worldwide: among the selected countries, highest rate of novel mutations (additional mutations) have been observed in USA followed by India and Spain (red color) and same is the case with S protein mutations (Blue color). A number of deletions were observed in Spain isolates followed by Italy and USA (Yellow color). Additionally, N protein mutations were also observed in all the isolates of SARS-COV-2 variants (Green color).
