# Supplementary material for: Mutations of SARS-CoV-2 Structural Proteins in the Alpha, Beta, Gamma, and Delta Variants: Bioinformatics Analysis
Source: JMIR Bioinform Biotechnol. 2023 Jul 14;4:e43906. doi: 10.2196/43906 (PMC10353769; doi:10.2196/43906)
Supplement: Multimedia Appendix 10 [file bioinform_v4i1e43906_app10.docx]

**
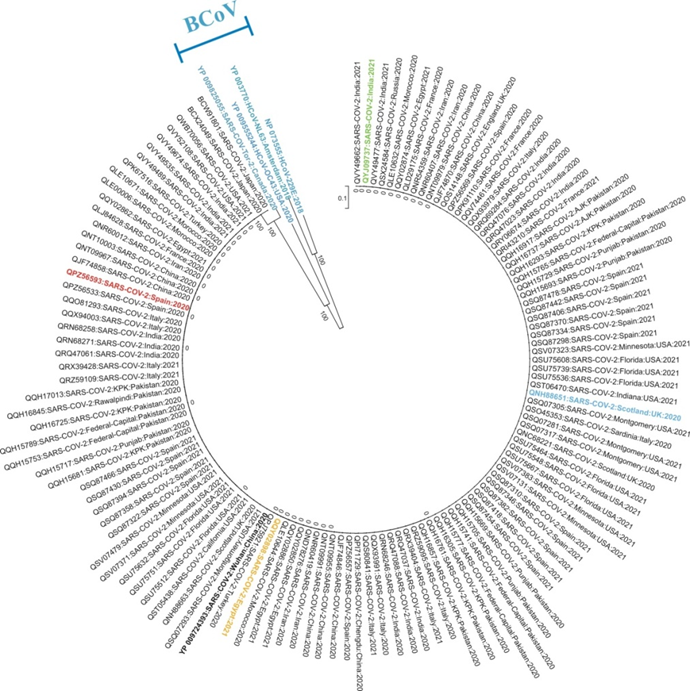
**

Neighbor-joining phylogenetic tree of M protein SARS-CoV-2: few mutations observed and each of the mutation is highlighted separately. Deletion at 72position in Spain (red), I82T mutation in India (green), E12X and F28X mutations in UK (blue), V70L mutation in Egypt (orange). Beta coronavirus strains used as out-group (dark blue color).

**
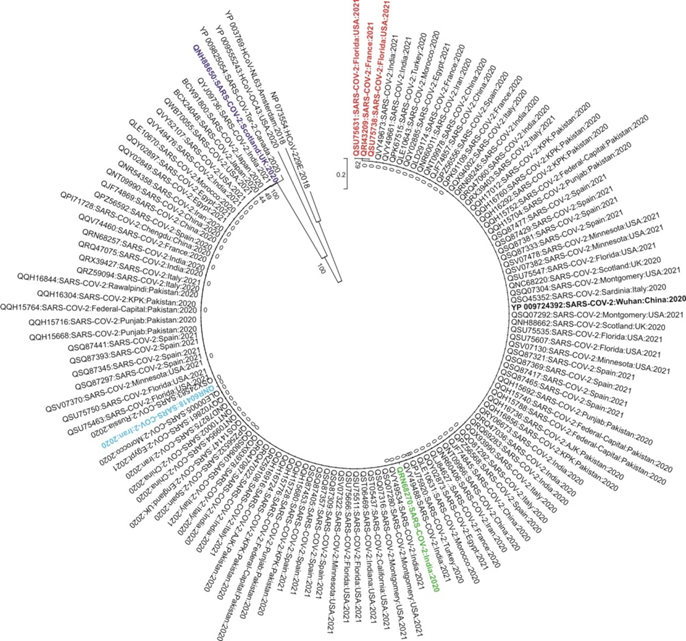
**

Neighbor-joining phylogenetic tree of E protein of SARS-CoV-2: few mutations observed and each of the mutation is highlighted separately. Detection of V58F in India (green), L28P mutation in Iran (blue), T30I and L51X mutations in UK (purple), P71L mutation in France and USA (red).
