# Supplementary material for: Mutations of SARS-CoV-2 Structural Proteins in the Alpha, Beta, Gamma, and Delta Variants: Bioinformatics Analysis
Source: JMIR Bioinform Biotechnol. 2023 Jul 14;4:e43906. doi: 10.2196/43906 (PMC10353769; doi:10.2196/43906)
Supplement: Multimedia Appendix 11 [file bioinform_v4i1e43906_app11.docx]

Novel mutations in S protein of SARS-CoV-2

| **Mutations** | **Amino acid Substitution** |
| --- | --- |
| **L1203R** | Leucine (L) was replaced by Arginine (R) at 1203position |
| **T732A** | Threonine (T) was replaced by Alanine (A) at 732position |
| **C488R** | Cysteine (C) was replaced by Arginine (R) at 488position |
| **L948I** | Leucine (L) was replaced by Isoleucine (I) at 948position |
| **S12F** | Lycine (S) was replaced by Phenylalanine (F) at 12position |
| **D627A** | Aspartic acid (D) was replaced by Alanine (A) at 627position |
| **T20I/N** | Threonine (T) was replaced by Isoleucine (I) or asparagine (N) at 20position |
| **T1117I** | Threonine (T) was replaced by Isoleucine (I) at 1117position |
| **G1251V** | Glycine (G) was replaced by Valine (V) at 1251position |
| **G769V** | Glycine (G) was replaced by Valine (V) at 796position |
| **Q613H** | Glutamine (Q) was replaced by Histidine (H) at 613position |
| **G219R** | Glycine (G) was replaced by Arginine (R) at 219position |
| **T22I** | Threonine (T) was replaced by Isoleucine (I) at 22position |
| **V367F** | Valine (V) was replaced by Phenylalanine (F) at 367position |
| **S477N** | Serine (S) was replaced by Asparagine (N) at 477position |
| **P26S/L** | Proline (P) was replaced by amino acid Serine(S) or Leucine (L) at 26position |
| **R190S** | Arginine (R) was replaced by Serine (S) at 190position |
| **D138Y** | Aspartic acid (D) was replaced Tyrosine (Y) at 138position |
| **H655Y** | Histidine (H) was replaced by Tyrosine (Y) at 655position |
| **T1027I** | Threonine (T) was replaced by Isoleucine (I) at 1027position |
| **V1176F** | Valine (V) was replaced by Phenylalanine (F) at 1176position |
| **Q1071H** | Glutamine (Q) was replaced by Histidine (H) at 1071position |
| **K1204R** | Lysine (K) was replaced by Arginine (R) at 1204position |
| **E156G** | Glutamic acid (E) was replaced by Glycine (G) at 156position |
| **A1020R** | Alanine (A) was replaced by Arginine (R) at 1020position |
| **E154K** | Glutamic acid (E) was replaced by Lysine (K) at 154position |
| **H1101D** | Histidine (H) was replaced by Aspartic acid (D) at 1101position |
| **T859I** | Threonine (T) was replaced by Isoleucine (I) at 859position |
| **Q677H/X** | Glutamine (Q) was replaced by Histidine (H) or unknown (X) at 677position |
| **S13I** | Serine (S) was replaced by Isoleucine (I) at 13position |
| **W152C** | Tryptophan (W) was replaced by Cysteine (C) at 152position |
| **S939F** | Serine (S) was replaced by Phenylalanine (F) at 939position |
| **S221L** | Serine (S) was replaced by Leucine (L) at 221position |
| **G257S** | Glycine (G) was replaced by Serine (S) at 257position |
| **Q414R** | Glutamine (Q) was replaced by Arginine (R) at 414position |
| **Q1207H** | Glutamine (Q) was replaced by Histidine (H) at 1207position |
| **D1163Y** | Aspartic acid (D) was replaced by Tyrosine (Y) at 1163position |
| **S813N** | Serine (S) was replaced by Asparagine (N) at 813position |
| **T778I** | Threonine (T) was replaced by Isoleucine (I) at 778position |
| **S868G** | Serine (S) was replaced by Glycine (G) at 868position, |
| **P681L/X** | acid Proline (P) was replaced by Leucine (L) or (X) at 681position |
| **H146R** | Histidine (H) was replaced by Arginine (R) at 146position, |
| **N1192S** | Asparagine (N) was replaced by Serine (S) at 1192position |
| **D808G** | Aspartic acid (D) was replaced by Glycine (G) at 808position |
| **F490S** | Phenylalanine (F) was replaced by Serine (S) at 490position |
| **S943P** | Serine (S) was replaced by Proline (P) at 943position |
| **L54F** | Leucine (L) was replaced by Phenylalanine (F) at 54position |

Novel mutations in N protein of SARS-CoV-2

| **Mutations** | **Amino acid Substitution** |
| --- | --- |
| **D63G** | Aspartic acid (D) was replaced by Glycine (G) at 63position |
| **H145Y** | Histidine (H) was replaced by Tyrosine (Y) at 145position |
| **G212V/X** | Glycine (G) was replaced by Valine (V) or unknown (X) at 212position |
| **G25X** | Glycine (G) was replaced by unknown (X) at 25position |
| **M210I** | Methionine (M) was replaced by Isoleucine (I) at 210position, |
| **Q418H** | Glutamine (Q) was replaced by Histidine (H) at 418position |
| **M317I** | Methionine (M) was replaced by Isoleucine (I) at 317position |
| **H300Y** | Histidine (H) was replaced by Tyrosine (Y) at 300position |
| **S186F** | Serine (S) was replaced by Phenylalanine (F) at 186position |
| **A376T** | Alanine (A) was replaced by Threonine (T) at 376position |
| **G215R** | Glycine (G) was replaced by Arginine (R) at 215position |
| **S197L** | Serine (S) was replaced by Leucine (L) at 197position |
| **S237X** | Serine (S) was replaced by unknown (X) at 237position |
| **I292T** | Isoleucine (I) was replaced by Threonine (T) at 292position |
| **P67S** | Proline (P) was replaced by Serine (S) at 67position |
| **P199L** | Proline was replaced by Leucine (L) at 199position |
| **L139F** | Leucine (L) was replaced by Phenylalanine (F) at 139position |
